# Positive selection analyses identify a single WWE domain residue that shapes ZAP into a more potent restriction factor against alphaviruses

**DOI:** 10.1371/journal.ppat.1011836

**Published:** 2024-08-29

**Authors:** Serina Huang, Juliana Girdner, LeAnn P. Nguyen, Carina Sandoval, Oliver I. Fregoso, David Enard, Melody M. H. Li

**Affiliations:** 1 Department of Human Genetics, David Geffen School of Medicine, University of California, Los Angeles, California, United States of America; 2 Department of Chemistry and Biochemistry, University of California, Los Angeles, California, United States of America; 3 Department of Microbiology, Immunology and Molecular Genetics, University of California, Los Angeles, California, United States of America; 4 Molecular Biology Institute, University of California, Los Angeles, California, United States of America; 5 Department of Ecology and Evolutionary Biology, University of Arizona, Tucson, Arizona, United States of America; 6 AIDS Institute, David Geffen School of Medicine, University of California, Los Angeles, California, United States of America; University of Colorado Denver, UNITED STATES OF AMERICA

## Abstract

The host interferon pathway upregulates intrinsic restriction factors in response to viral infection. Many of them block a diverse range of viruses, suggesting that their antiviral functions might have been shaped by multiple viral families during evolution. Host-virus conflicts have led to the rapid adaptation of host and viral proteins at their interaction hotspots. Hence, we can use evolutionary genetic analyses to elucidate antiviral mechanisms and domain functions of restriction factors. Zinc finger antiviral protein (ZAP) is a restriction factor against RNA viruses such as alphaviruses, in addition to other RNA, retro-, and DNA viruses, yet its precise antiviral mechanism is not fully characterized. Previously, an analysis of 13 primate ZAP orthologs identified three positively selected residues in the poly(ADP-ribose) polymerase-like domain. However, selective pressure from ancient alphaviruses and others likely drove ZAP adaptation in a wider representation of mammals. We performed positive selection analyses in 261 mammalian ZAP using more robust methods with complementary strengths and identified seven positively selected sites in all domains of the protein. We generated ZAP inducible cell lines in which the positively selected residues of ZAP are mutated and tested their effects on alphavirus replication and known ZAP activities. Interestingly, the mutant in the second WWE domain of ZAP (N658A) is dramatically better than wild-type ZAP at blocking replication of Sindbis virus and other ZAP-sensitive alphaviruses due to enhanced viral translation inhibition. The N658A mutant is adjacent to the previously reported poly(ADP-ribose) (PAR) binding pocket, but surprisingly has reduced binding to PAR. In summary, the second WWE domain is critical for engineering a more potent ZAP and fluctuations in PAR binding modulate ZAP antiviral activity. Our study has the potential to unravel the role of ADP-ribosylation in the host innate immune defense and viral evolutionary strategies that antagonize this post-translational modification.

## Author summary

Host proteins and viral proteins that encounter one another are locked in a perpetual genetic arms race. In this evolutionary race, a mutation that confers a survival advantage will become more frequent in the population. By looking at the sequences of genes that are known to have antiviral roles in mammals, we can identify the exact sites where a host and viral protein have interacted and gain insight into how an antiviral protein works. Here, we identified these sites in zinc finger antiviral protein (ZAP), a host protein that blocks many different viruses. We found that changing one of the sites from the original amino acid to another dramatically improves ZAP’s antiviral activity against Sindbis virus, an alphavirus, due to improved inhibition of viral translation. Our mutation is also better at inhibiting other members in the *Alphavirus* genus. We observed that our mutant ZAP has reduced ability to bind poly(ADP-ribose), a post-translational modification that is targeted by alphaviruses for productive infection. Our findings help us better understand how viruses have shaped the evolution of broad-spectrum host antiviral proteins, with great implications for the engineering of super restriction factors.

## Introduction

Host and viral proteins are constantly engaging in genetic conflicts that create selective pressures on the other side to evolve. In a host immune protein, an advantageous mutation that successfully maintains recognition of a viral protein or evades a viral antagonist will rise in frequency, a phenomenon called positive selection. The amino acid sites on which positive selection has acted can be identified by bioinformatic approaches when the non-synonymous substitution rate is estimated to exceed the synonymous substitution rate [1,2]. The signatures of positive selection on a protein can inform us about historical interaction hotspots between the host and virus [3], as well as highlight sites that have important antiviral roles in winning the host-virus arms race.

Signatures of positive selection are especially prevalent in host interferon (IFN)-stimulated genes (ISGs) that are induced to counteract viral infections [3]. One of these ISGs is zinc finger antiviral protein (ZAP), also known as poly(ADP-ribose) polymerase 13 (PARP13) [4]. ZAP inhibits a diverse range of virus genera, yet its antiviral activity can be specific to particular members in a genus, suggesting viral evasion or antagonism of ZAP inhibition [5,6]. For example, ZAP blocks many species of mosquito-borne alphaviruses to varying degrees, where Sindbis virus (SINV) and Ross River virus (RRV) are more sensitive than o’nyong’nyong virus (ONNV) and chikungunya virus (CHIKV) vaccine strain 181/clone 25 [7,8]. Alphaviruses have a positive-sense RNA genome, which can be immediately translated into viral proteins by host ribosomes upon entry into the host cell [9,10]. The viral proteins then replicate the viral genome, leading to the production of structural proteins and the assembly of mature virus particles. It is in the early stages of infection that ZAP acts to prevent the translation of alphaviral RNA by synergizing with the host E3 ubiquitin ligase, tripartite motif containing 25 (TRIM25) [11,12].

ZAP has two major splice isoforms, ZAPS (short) and ZAPL (long), with distinct antiviral and immunomodulatory activities [7,13–15]. Recently discovered isoforms ZAPM (medium) and ZAPXL (extralong) resemble the antiviral activities of ZAPS and ZAPL, respectively [7]. The N-terminus of ZAP contains four zinc fingers (ZnFs) that bind RNA. It is followed by a central region that consists of a fifth ZnF and two WWE domains, named for the WWE motif containing tryptophan, tryptophan, and glutamic acid. The ADP-ribose-binding ability of the second WWE domain (WWE2) has only been recently discovered [16,17]. At the C-terminus, ZAPL has a PARP-like domain that is catalytically inactive and cannot ADP-ribosylate substrates [18,19], but confers more antiviral activity on the longer isoforms [7,15,20,21]. Even though the RNA binding activity of ZAP has been extensively studied, the manner in which the other domains contribute to ZAP’s antiviral activity is not well characterized.

While ZAP has been shown to be positively selected [15,22], there are outstanding questions about the antiviral mechanism of ZAP and how its cellular functions contribute to viral inhibition. A previous study performed positive selection analysis on ZAP sequences from 13 primate species and found three positively selected sites, all in the PARP-like domain. However, limiting positive selection analyses to primate ZAP sequences only identifies sites that have been selected for rapid adaptation throughout primate evolution. While primates are thought to be the natural hosts of HIV and simian immunodeficiency viruses [23], ZAP has broad-spectrum antiviral activity against diverse viruses which infect a wider range of mammals (e.g. alphaviruses, flaviviruses, coronaviruses to name a few). Thus, we inferred that other mammalian ZAP orthologs must have also faced selective pressure from this host-virus arms race. By restricting positive selection analyses to only primate ZAP, one might miss positive selection signals contributed by non-primate species. ZAP has a long-standing history of host-virus interactions and likely arose from a gene duplication event after the divergence of tetrapods [24]. Assuming that at least some of the positively selected sites are driven by the ancestors of extant ZAP-sensitive viruses, we would expect to detect positive selection signals from a broader range of mammals which these viruses tend to infect.

Here, we performed positive selection analyses on 261 mammalian ZAP sequences using four complementary and sophisticated models that make more realistic assumptions about the substitution rates. We identified seven residues that are positively selected in ZAP, most of which are outside the PARP-like domain. We mutated each positively selected site and found that one mutant in the WWE2 (N658A) has antiviral activity that is almost 10 times stronger than wild-type (WT) ZAP against SINV, creating a restrictor that is more antiviral than any versions of ZAP that were previously reported. The N658A mutant is more efficient than ZAPL WT at inhibiting virion production of SINV and replication of a panel of alphaviruses in a manner that is dependent on viral translation suppression. Interestingly, mutation of both positively selected sites in the WWE2 that form a potential interaction surface does not further increase the antiviral activity of ZAP.

We then investigated the role of viral RNA binding, TRIM25 interaction, IFN response, and poly(ADP-ribose) (PAR) binding in mediating the activity of a more potent restrictor ZAP. We found that the superior antiviral activity of the N658A mutant is correlated with changes in PAR binding by the ZAPL mutant. We mutated site 658 to orthologous residues found in other mammalian species and observed that none of them is as antiviral as the N658A mutant. This surprising finding suggests that evolutionary forces did not steer human ZAP to be the most antiviral, at least not against alphaviruses. By taking into account the history of host-virus conflicts, positive selection analyses allow us to identify specific sites with high impact on the effectiveness of the host antiviral program, providing a blueprint for generating stronger restriction factors.

## Results

### ZAP is positively selected throughout mammalian evolution at novel sites

We used the longest isoform of ZAP, ZAPXL, to curate and align 261 high quality mammalian orthologs. We ran four positive selection tests with complementary strengths on the alignment of mammalian ZAP sequences: Fixed Effects Likelihood (FEL); Mixed Effects Model of Evolution (MEME); Fast, Unconstrained Bayesian AppRoximation (FUBAR); and the Bayesian mutation-selection model by Rodrigue *et al* [25–28]. FEL does not make assumptions about the distribution of selection parameters over sites but assigns independent non-synonymous and synonymous rates to each site. MEME accounts for the fact that positive selection occurs episodically, rather than remaining constant over time. FUBAR improves upon random effect likelihood models [29] by implementing more parametrically complex models. Rodrigue *et al*.’s method is the first Bayesian mutation-selection model, offering higher sensitivity.

**Fig 1:**
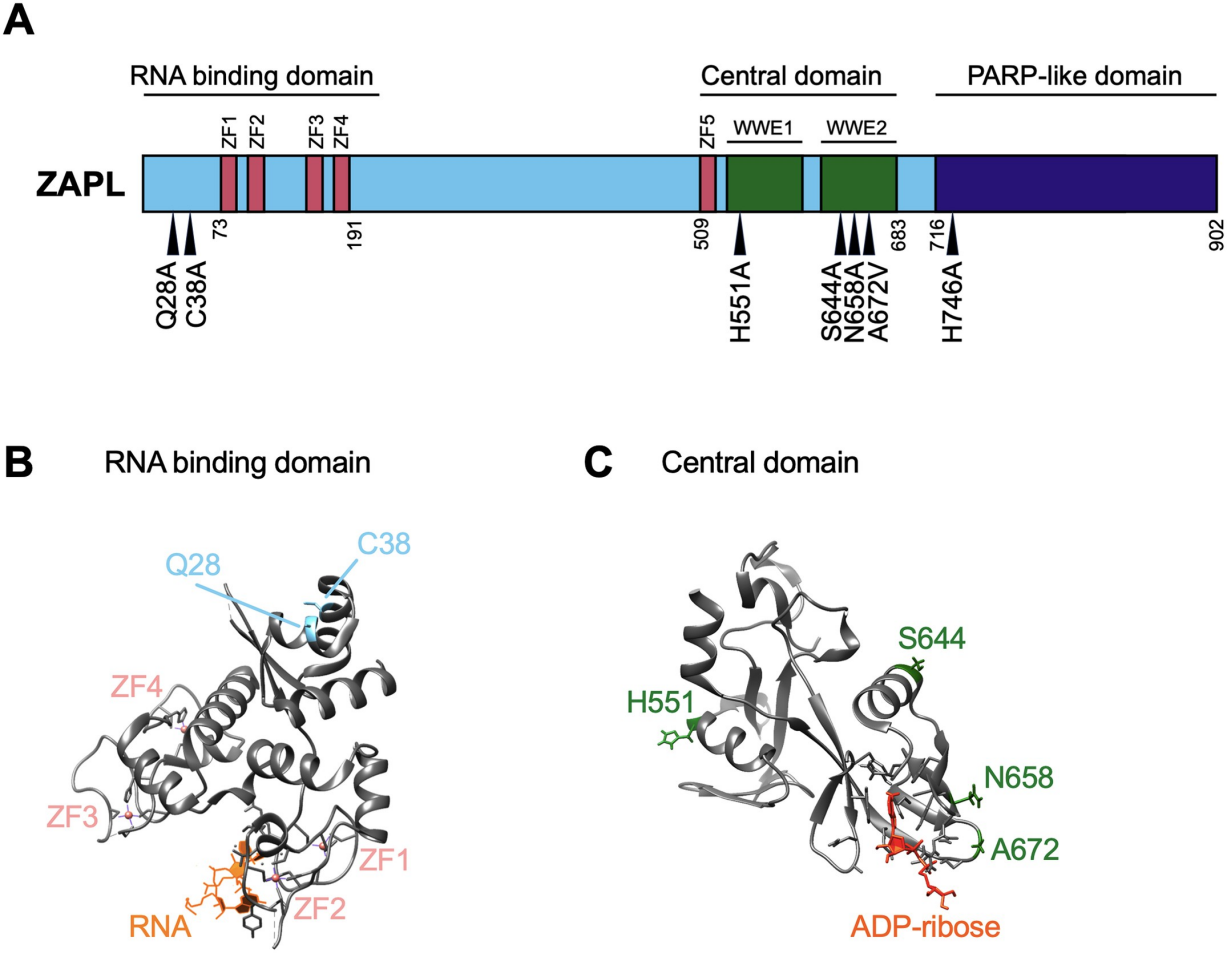
Identification of seven positively selected sites across ZAP protein. (A) A schematic of the ZAPL isoform annotated with its domains. Triangles indicate positively selected sites identified from the overlap of four methods: Fixed Effects Likelihood; Mixed Effects Model of Evolution; Fast, Unconstrained Bayesian AppRoximation; and the Bayesian mutation-selection model by Rodrigue *et al*. [25–28]. (B) ZAP RNA binding domain bound to RNA. The structure (PDB: 6UEJ) [30] was visualized with UCSF Chimera [77]. Positively selected Q28 and C38 residues shown in blue; RNA in orange; zinc fingers in salmon. (C) ZAP central domain bound to ADP-ribose. The structure (PDB: 7TGQ) [17] was visualized with UCSF Chimera. Positively selected sites H551, S644, N658, and A672 shown in green; ADP-ribose in dark orange.

To validate the robustness of our tests, we ran the 13 primate ZAP sequences from the study by Kerns *et al*. [15] and were able to replicate the three positively selected sites previously identified. Using the 261 mammalian ZAP, we identified seven positively selected sites that are shared by all four tests (S1A Fig) and mapped them to human ZAP isoforms (S1B Fig). For consistency, the positively selected sites are numbered in the context of ZAPS and ZAPL, which are the better studied isoforms with antiviral activities similar to ZAPM and ZAPXL, respectively. The positively selected sites we identified are concentrated in specific regions spanning across the ZAP gene (Fig 1A). Two of these sites are within the first 254 amino acids of the protein, which comprise the RNA binding domain that is necessary for ZAP recognition and inhibition of viral RNA. These residues, Q28 and C38, are relatively close to each other but are positioned opposite the RNA binding groove, with both of their side chains pointing away from the rest of the structure [30] (Fig 1B). RNA binding is essential to ZAP’s antiviral activity against murine leukemia virus [31], CpG-enriched HIV-1 [30], and SINV [32,33]. However, the identification of these two sites raises the possibility that viral proteins can interact with ZAP at a different location in its N-terminal region without interfering with binding to viral RNA.

More than half of the positively selected sites are in the central domain, three of which are tightly clustered in the WWE2, which has only recently been found in ZAP to bind PAR. When mapped to the available crystal structure of the central region consisting of the fifth zinc finger and the two WWE domains [16,17], two of the sites, N658 and A672, are next to the PAR binding pocket and face outward, suggesting that there is space to be accessed by viral proteins (Fig 1C). Taken together, our positive selection analyses demonstrate that ZAP has been rapidly evolving not just during primate evolution, but also during mammalian evolution. These novel positively selected residues in ZAP are found in all domains of ZAP, suggesting that ancient viruses have likely targeted and antagonized ZAP at distinct sites.

### One of the positively selected site mutants we generated affects ZAP antiviral phenotype against SINV

To probe the effect of the positively selected sites, we mutated each site from the WT amino acid in humans to alanine because alanine is chemically inert and would not dramatically change the secondary structure of the protein [34]. In the case where the WT amino acid is alanine, we mutated it to valine, the next closest amino acid. We cloned either WT or mutant ZAPS and ZAPL with an N-terminal 3XFLAG tag into the ePiggyBac (ePB) transposon system and generated stable cell lines in ZAP knockout (KO) HEK293T cells (S2 Fig) [35,36]. We tested the mutants in the ZAPS and ZAPL background because ZAPS and ZAPL are most commonly studied and have comparable antiviral activities to ZAPM and ZAPXL, respectively.

**Fig 2:**
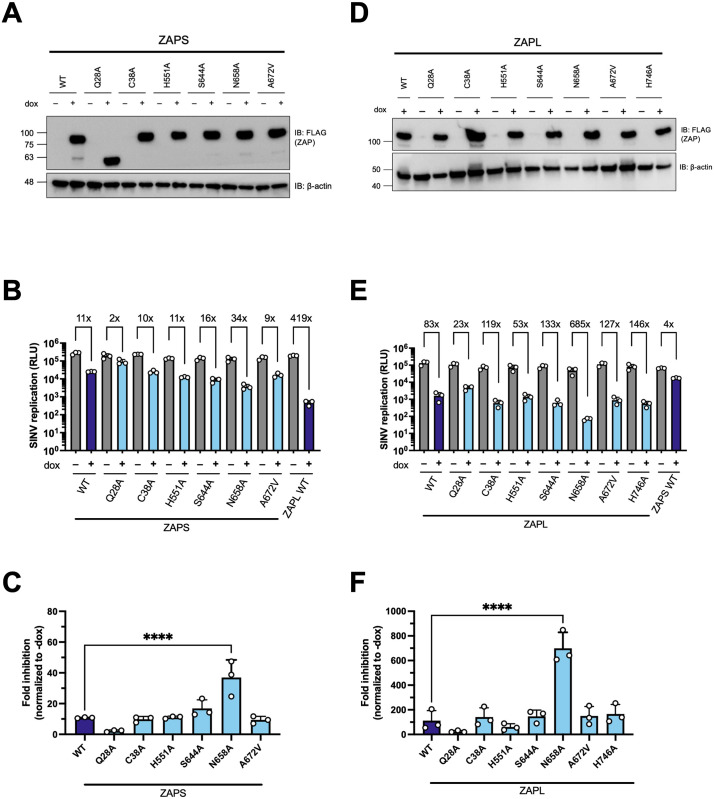
Each positively selected site in ZAP was mutated and its antiviral activity against Sindbis virus (SINV) was tested. (A, D) Western blot of (A) ZAPS or (D) ZAPL wild-type (WT) or positive selection mutants inducible ZAP knockout (KO) HEK293T whole cell lysates (WCL). (B, E) (B) ZAPS or (E) ZAPL WT or mutant ZAP KO HEK293T cells were induced for ZAP expression for 24 hours before infection with SINV Toto1101/Luc at a multiplicity of infection (MOI) of 0.01 plaque forming units (PFU)/cell and harvested at 24 hours post-infection (h.p.i.) for luciferase assay by measuring relative luciferase units (RLU). Data are representative of two independent experiments. 1μg/mL doxycycline (dox) is used to induce ZAP expression. (C, F) Fold inhibition values of each ZAPS (C) and ZAPL (F) cell line are calculated by dividing the averaged -dox RLU by the individual +dox RLU. The averaged fold inhibition for each cell line is shown on top of the bars in (B) and (E). Error bars indicate standard deviation. Asterisks indicate statistically significant differences as compared to the corresponding WT cell line (one-way ANOVA and Bartlett’s test: ****, p<0.0001).

Almost all the mutant cell lines have robust ZAP expression when induced by doxycycline (dox) (Fig 2A and 2D), with the exception of ZAPS Q28A which appears to have a truncation at the C-terminus, as it is still able to be detected by the N-terminal FLAG tag (Fig 2A). Since our candidate sites are positively selected throughout mammalian evolution, we chose to test their antiviral activity against alphaviruses, whose primary hosts are mammals such as primates, horses, and rodents [37]. We first infected the ZAP cell lines with SINV, a prototype alphavirus that is susceptible to ZAP inhibition.

We infected ZAPS and ZAPL WT and mutant cell lines with a luciferase-expressing SINV reporter virus. To quantify the antiviral activity, we divided the averaged -dox values by the individual triplicate +dox values in each cell line to get three fold inhibition values. Despite differences in absolute fold inhibitions between independent experiments featuring ZAPS and ZAPL mutants, we found that ZAPL WT is invariably more antiviral than ZAPS WT, consistent with previous reports [7,15]. While a couple of mutants have lower fold inhibition than WT ZAP, others have higher fold inhibition (Fig 2B and 2E), though they are not statistically significant. Notably, the N658A mutant located in the WWE2 shows a statistically significant improvement in ZAP antiviral activity than the WT (Fig 2C and 2F, three times better than ZAPS WT and eight times better than ZAPL WT). In addition, some mutants displayed isoform-specific effects. For instance, ZAPL C38A has higher fold inhibition than ZAPL WT, but its ZAPS counterpart is similarly antiviral to ZAPS WT. Densitometric quantification of the amount of ZAP in each cell line seems to have no correlation with anti-SINV activity (S3 Fig). These results suggest that altering the naturally occurring amino acid at a positively selected site *a posteriori* changes the antiviral activity of ZAP against SINV and that adaptations at a site can have important functional consequences.

**Fig 3:**
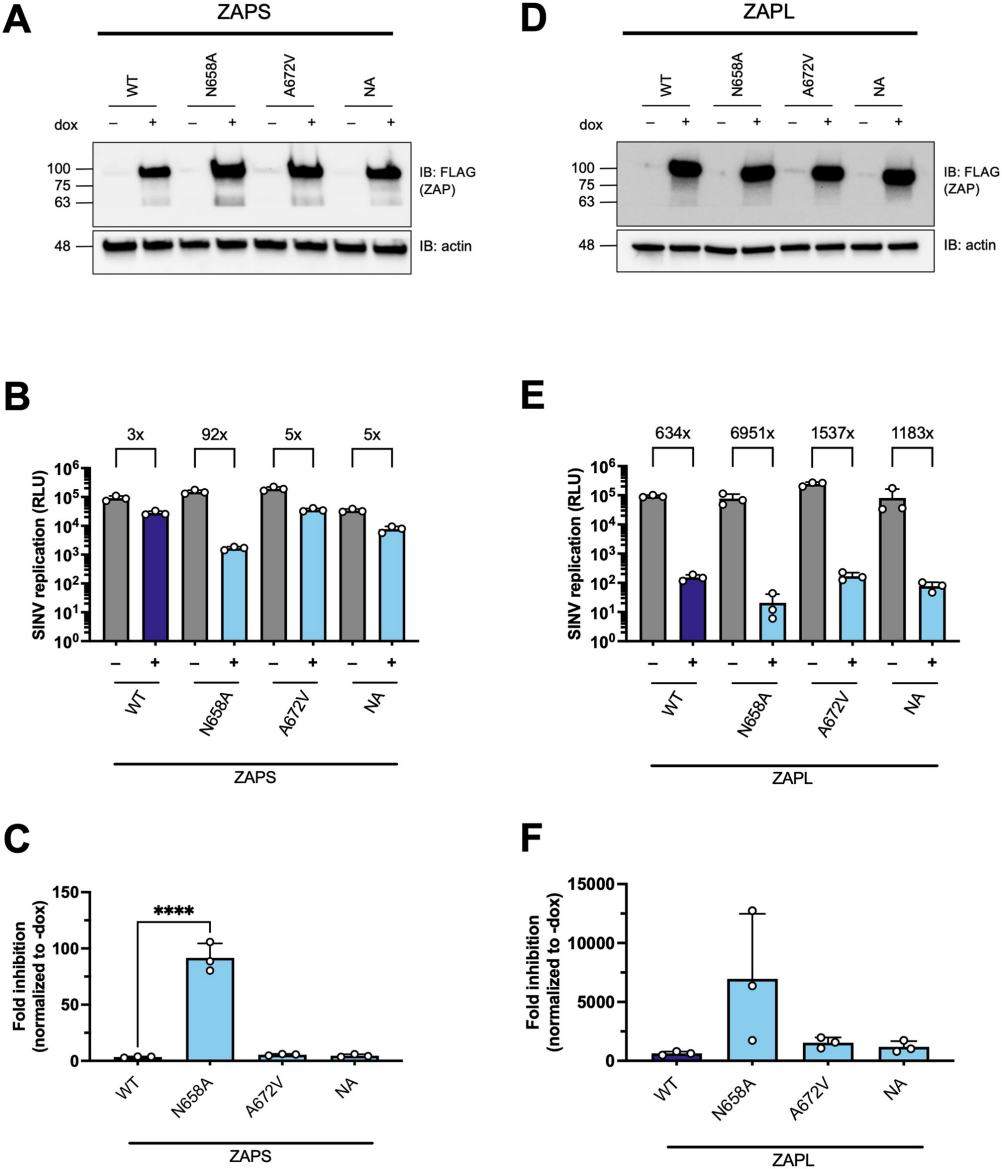
Mutating both positively selected sites in the second WWE domain of ZAP does not further enhance antiviral activity. (A, D) Western blot of (A) ZAPS or (D) ZAPL WT, N658A, A672V, or N658A/A672V (NA) double mutant inducible ZAP KO HEK293T cell lysates. (B, E) (B) ZAPS or (E) ZAPL WT or mutant ZAP KO HEK293T cells were induced for ZAP expression for 24 hours before infection with SINV Toto1101/Luc at an MOI of 0.01 PFU/cell and harvested at 24 h.p.i for luciferase assay. Data are representative of three (B) and three out of four (E) independent experiments. 1μg/mL dox is used to induce ZAP expression. (C, F) Fold inhibition values of each ZAPS (C) and ZAPL (F) cell line are calculated by dividing the averaged -dox RLU by the individual +dox RLU. The averaged fold inhibition for each cell line is shown on top of the bars in (B) and (E). Error bars indicate standard deviation. Asterisks indicate statistically significant differences as compared to the corresponding WT cell line (one-way ANOVA and Dunnett’s test: ****, p<0.0001).

Since both sites 658 and 672 are located in the WWE2 and flank the PAR binding pocket in the crystal structure (Fig 1A and 1C), we wondered if site 672 can bolster the superior antiviral effect of site 658, as in the case with TRIM5α [38]. We generated the double mutant N658A/A672V (NA) in the same ZAP KO ePB system and assessed its ability to restrict SINV replication. Both ZAPS and ZAPL NA double mutants are as stably expressed as the single mutants (Fig 3A and 3D). To our surprise, the antiviral activity of the ZAPS NA double mutant is not an intermediate between ZAPS N658A and A672V; rather, it diminishes the antiviral activity of N658A to that of ZAPS WT and A672V (Fig 3B and 3C), suggesting that A672V may have a dominant negative effect on N658A in ZAPS. The ZAPL NA double mutant likewise does not approach the strength of ZAPL N658A (Fig 3E and 3F). The differential antiviral activity of the A672V single mutant and the NA double mutant in ZAPS and ZAPL again highlights isoform specificity at particular sites. Together, the WWE2 mutations in combination lessen the increase in antiviral activity we observed with the single N658A mutation in both ZAPS and ZAPL backgrounds, suggesting that these mutations may not act as a single protein interaction surface.

### The ZAPL N658A mutant blocks the early steps of alphaviral infection more effectively

**Fig 4:**
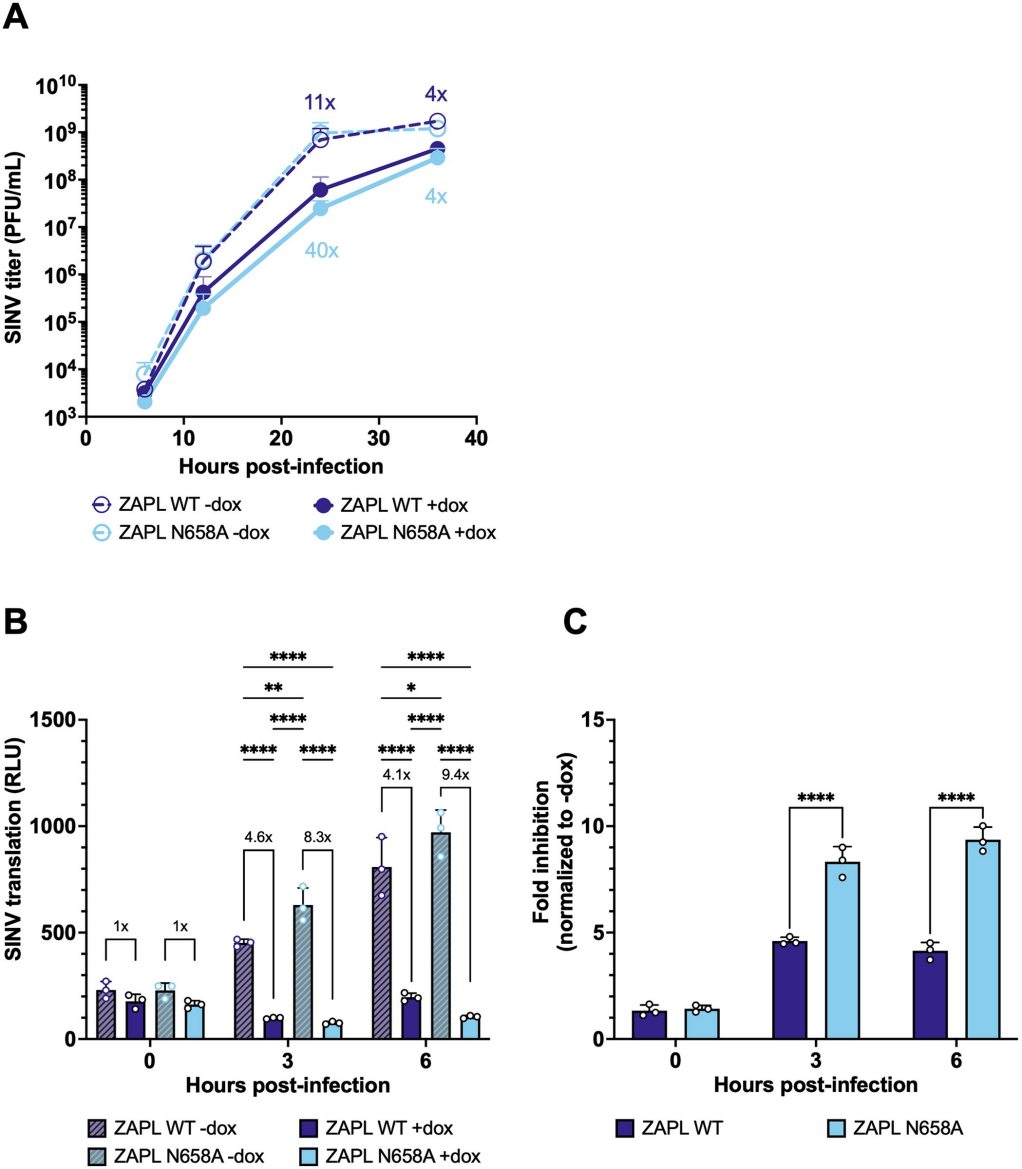
The N658A mutant is better at inhibiting virion production and SINV RNA translation. ZAPL WT or N658A ZAP KO HEK293T cells were induced for ZAP expression with 1μg/mL dox 24 hours prior to infection. Cells were infected with (A) SINV Toto1101 at an MOI of 0.01 PFU/cell, harvesting supernatant at 6, 12, 24, and 36 h.p.i. for plaque assays. Viral titers of plaque assays are determined in BHK-21 cells. Data are combined from two independent experiments. Fold inhibition values of each cell line are calculated by dividing the averaged -dox titer by the individual +dox titer. The averaged fold inhibition for each cell line is shown on top of the bars. Error bars indicate standard deviation; or (B, C) SINV Toto1101/Luc:ts6 at an MOI of 0.01 PFU/cell, and harvested at 0, 3, and 6 h.p.i. for luciferase assay. Data are representative of two independent experiments. Fold inhibition values of each cell line are calculated by dividing the averaged -dox RLU by the individual +dox RLU. The averaged fold inhibition for each cell line is shown on top of the bars in (B). Error bars indicate standard deviation. Asterisks indicate statistically significant differences as compared to every other condition at each timepoint (B, two-way ANOVA and Tukey’s multiple comparisons test: *, p<0.05; **, p<0.01; ***, p<0.001; ****, p<0.0001) or compared to the WT cell line (C, two-way ANOVA and Šídák’s multiple comparisons test: ****, p<0.0001).

We were interested by the superior antiviral activity of the N658A mutant alone and focused on the ZAPL isoform to study the mutant in the presence of all domains of ZAP, including the PARP-like domain. We wanted to determine whether the effects on viral replication impact the overall virion production. We infected ZAPL WT or N658A cells with SINV and collected the cell supernatant containing mature and released virions at 0, 6, 12, 24, and 36 hours post-infection (h.p.i.). We determined the viral titer on BHK-21 cells via plaque assay. We found that both ZAPL WT and N658A inhibited SINV virion production, but at 24 h.p.i., ZAPL N658A is about 4-fold more inhibitory (Fig 4A, 11x vs. 40x), consistent with the phenotype we observed with viral replication.

Next, we sought to determine the stage in the viral life cycle at which the ZAPL N658A mutant acts. Because ZAP is known to act by blocking alphaviral RNA translation, we tested the positively selected ZAP mutant N658A against a temperature-sensitive replication-deficient SINV luciferase reporter virus [39]. The temperature-sensitive SINV luciferase reporter virus (ts6 mutant) has a single glycine to glutamine mutation in the viral RNA-dependent RNA polymerase [40] and is therefore unable to replicate the viral genome at the non-permissive temperature (40°C) at which infection was carried out. As a result, only the incoming viral genomic RNA is translated. We infected ZAP WT and N658A cell lines with the replication-deficient virus at the non-permissive temperature and found that the N658A mutant is about two times better at blocking SINV RNA translation at 3 h.p.i. and 6 h.p.i. (Fig 4B and 4C), which is a difference that is statistically significant. Our finding supports that the superior antiviral activity of the N658A mutant is likely due to an enhanced block at the step of incoming viral RNA translation.

**Fig 5:**
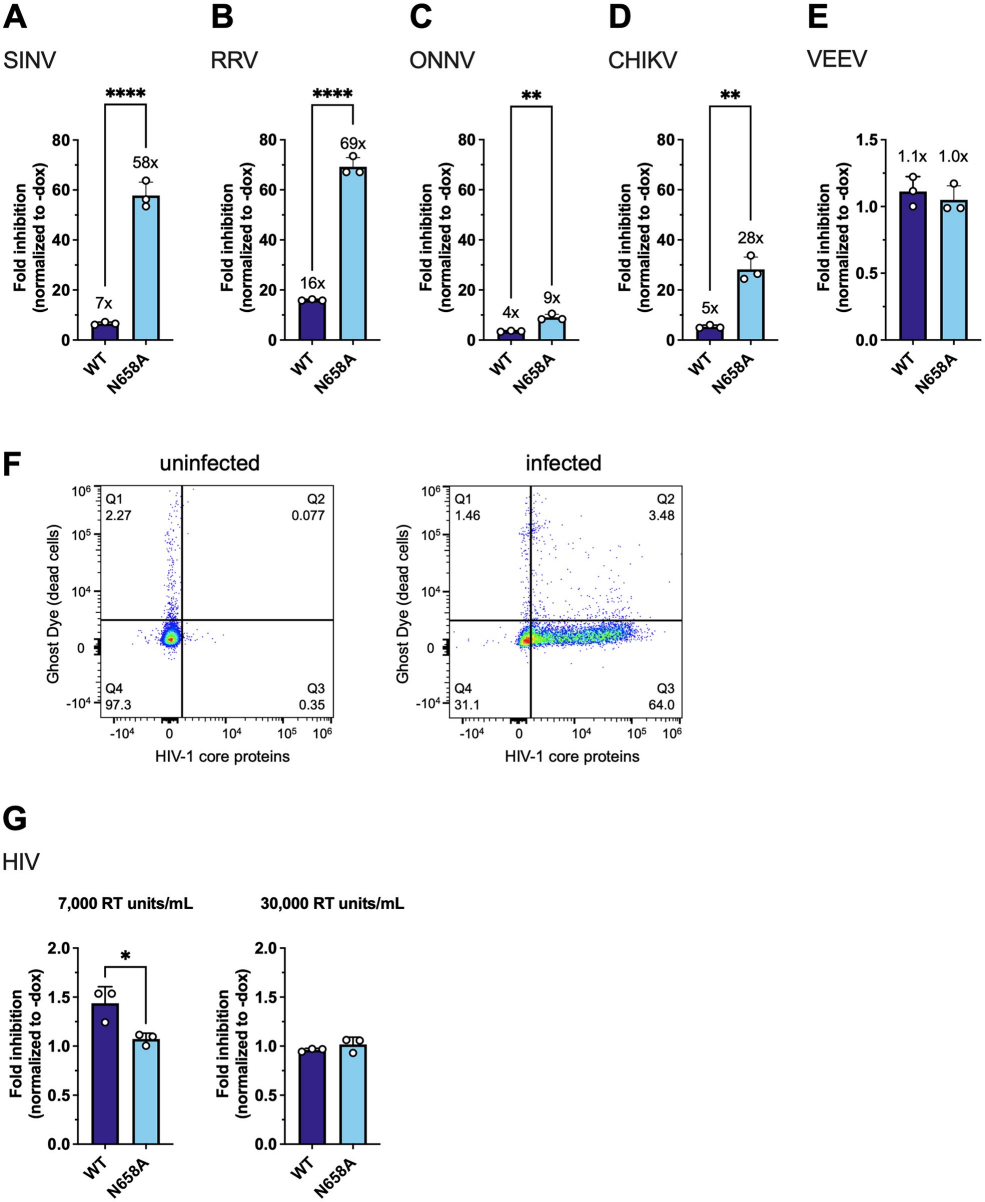
The ZAPL N658A mutant inhibits many other alphaviruses better than WT. After 24 hours of 1μg/mL dox treatment, ZAPL WT or N658A ZAP KO HEK293T cells were infected with (A) GFP-expressing Sindbis virus (SINV, MOI = 0.01), (B) Ross River virus (RRV, MOI = 1), (C) o’nyong’nyong virus (ONNV, MOI = 1), (D) chikungunya virus (CHIKV, MOI = 0.1), or (E) Venezuelan equine encephalitis virus (VEEV, MOI = 0.1) PFU/cell for 24 hours before their percentage of infection was determined by flow cytometry. Data are representative of at least two independent experiments of biological replicates in triplicate wells. Fold inhibition values of each cell line are calculated by dividing the averaged -dox % GFP infection by the individual +dox % GFP. The averaged fold inhibition for each cell line is shown on top of the bars. Error bars indicate standard deviation. Asterisks indicate statistically significant differences as compared to the WT cell line (unpaired t-test: **, p<0.01; ****, p<0.0001). (F, G) Following 24 hours of 1μg/mL dox treatment, ZAPL WT or N658A ZAP KO HEK293T cells were spinfected by an HIV-1 isolate BRU ΔEnv pseudotyped with the vesicular stomatitis virus glycoprotein at 7,000 or 30,000 reverse transcriptase (RT) units/mL. 24 hours later, the cells were analyzed for the percentage of infection (HIV-1 core antigen) and viability (Ghost Dye) via flow cytometry. Flow cytometry plots of an uninfected (left) and infected (right) sample (F). The fold inhibition is calculated by normalizing the percentage of infection in the +dox samples to the averaged percentage of infection in the corresponding -dox samples (G). Data are representative of two independent experiments of biological replicates in triplicate wells. Error bars indicate standard deviation. Asterisks indicate statistically significant differences (Unpaired t-test: *, p<0.05).

Since we hypothesized that the positive selection of ZAP may be driven by ancient alphavirus-like viruses, we tested whether the N658A mutant also inhibits other alphaviruses better. We infected the ZAPL WT or N658A cell line with GFP-expressing SINV, RRV, ONNV, CHIKV vaccine strain 181/clone 25, and Venezuelan equine encephalitis virus (VEEV). Alphaviruses known to be more sensitive to ZAP inhibition are more inhibited by the N658A mutant (Fig 5A, 7x vs. 58x against SINV; Fig 5B, 16x vs. 69x against RRV), while the ones that are less sensitive [7,39] are similarly resistant to both ZAPL WT and N658A (Fig 5C, 4x vs. 9x against ONNV; Fig 5E, 1.1x vs. 1.0x against VEEV). Interestingly, even though we previously observed that the non-reporter CHIKV vaccine strain is less susceptible to ZAP inhibition [7], we saw that both ZAPL WT and N658A dramatically inhibited GFP-expressing CHIKV vaccine strain, with the N658A mutant being more antiviral than WT (Fig 5D). Since the CHIKV strain we tested expresses the GFP reporter under the control of the viral subgenomic promoter, our results suggest that ZAP might inhibit step(s) at or prior to viral subgenomic mRNA expression. The smaller difference in virion production between WT and N658A ZAP might be partly due to the fact that by the time we assay for virion production, there are many steps in the virus life cycle post-ZAP restriction for the virus to “catch up.” On the other hand, luciferase- and GFP-expressing alphaviruses have allowed us to see the effect of ZAP at isolated, specific steps leading up to viral RNA replication, where ZAP exerts its strongest effect during viral RNA translation.

As a broad-spectrum antiviral protein, it is very likely that ZAP has to balance its inhibitory activity against one virus at the expense of its inhibitory activity against other viruses. To test this evolutionary hypothesis, we infected our ZAPL WT and N658A mutant cell lines with HIV-1 Bru ΔEnv, a single-round infection virus that is deficient in the viral envelope gene and pseudotyped with the glycoprotein from vesicular stomatitis virus which infects broad cell types. We measured infection via flow cytometry and confirmed that more than 95% of the infected cells were viable (negative for the Ghost Dye stain), and gated for infected cells using an antibody against HIV-1 core proteins (Fig 5F). With a lower virus input of 7,000 reverse transcriptase (RT) units/mL, WT ZAP exhibits weak anti-HIV-1 activity (~1.5-fold inhibition) while the N658A mutant does not (~1-fold inhibition) (Fig 5G). Even though there is statistical significance between WT and N658A ZAP against HIV-1 at lower infection, the difference is minimal. With a higher virus input (30,000 RT units/mL), neither WT nor N658 ZAP inhibits HIV-1 replication. Taken together, these results show that the N658A mutant is not better than WT ZAP at inhibiting HIV-1.

### The improved antiviral activity of the N658A mutant is not due to changes in binding to SINV RNA, interaction with TRIM25, or increased activation of ISGs

**Fig 6:**
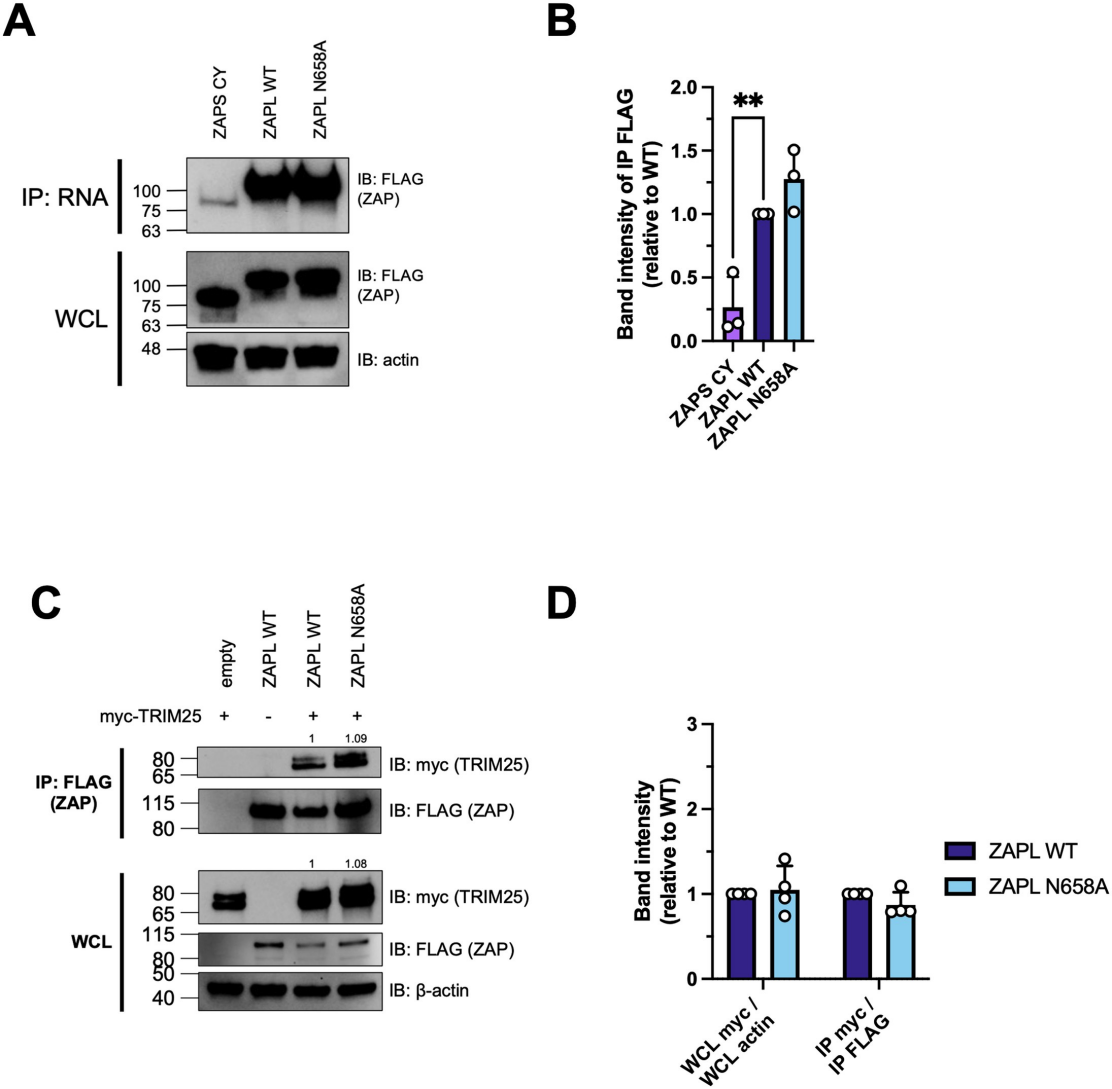
The improved antiviral activity of the N658A mutant is not due to changes in ZAP binding to SINV RNA or interaction with TRIM25. (A) Western blot of ZAPL CY, WT, or N658A in ZAP inducible ZAP KO HEK293T cell lysates bound to biotinylated SINV genomic RNA immunoprecipitated by streptavidin beads. Data are representative of three independent experiments. (B) Densitometric analysis of the amount of FLAG-ZAP immunoprecipitated by equal amounts of SINV RNA as quantified by Image Lab, normalized to WT, and combined from three independent experiments. Error bars indicate standard deviation. Asterisks indicate statistically significant differences as compared to the WT cell line (one-way ANOVA: **, p<0.01). (C) Western blot of TRIM25 bound to ZAP in cell lysates of ZAP KO HEK293T transfected with pcDNA3.1-3XFLAG-ZAPL and pcDNA3.1-myc-TRIM25. Lysates were immunoprecipitated by FLAG beads. Data are representative of four independent experiments. (D) Densitometric analysis of the amount of WCL myc-TRIM25 normalized to β-actin, and of the amount of myc-TRIM25 pulldown normalized to FLAG-ZAP pulldown as quantified by Image Lab, normalized to WT, and combined from four independent experiments. Error bars indicate standard deviation. Asterisks indicate statistically significant differences as compared to the WT cell line (two-way ANOVA and Dunnett’s multiple comparisons test).

To determine the mechanism of the enhanced antiviral activity of the N658A mutant, we characterized the mutant in terms of known abilities of ZAP. Since ZAP is recognized as a sensor of CpG-rich viral RNA, we wondered if N658A binds better to SINV genomic RNA than ZAPL WT does. We performed an *in vitro* RNA pulldown assay by incubating protein lysates from either the ZAPL WT or N658A cell line with equal amounts of biotinylated SINV genomic RNA. We pulled down the biotinylated viral RNA using streptavidin beads and probed for ZAP. We generated and tested a ZAP KO HEK293T cell line with inducible expression of a ZAPS C86A/Y96A mutant (ZAPS CY), which is deficient in RNA binding [32,33], as negative control. As expected, markedly less ZAPS CY is bound to equal amounts of SINV RNA compared to ZAPL WT (Fig 6A). Similar amounts of ZAPL WT and ZAPL N658A are bound to SINV RNA (Fig 6A). Averaged across three independent trials, a slightly higher amount of ZAPL N658A was bound to SINV RNA compared to ZAPL WT, but the difference was minimal (1.3x vs. 1x) and was not statistically significant (Fig 6B). Our results suggest that factors other than viral RNA binding may contribute to the enhanced antiviral activity of the mutant.

We then asked whether the N658A mutant changes ZAP’s ability to interact with TRIM25, a host E3 ubiquitin ligase that is a requisite cofactor for ZAP’s inhibition of SINV RNA translation [11,12]. We transfected 3XFLAG-ZAPL and myc-TRIM25 into ZAP KO HEK293T cells and performed a co-immunoprecipitation assay with FLAG beads. We found that ZAPL WT and N658A interact with TRIM25 similarly (Fig 6C). When we quantified the amount of overall myc-TRIM25 in the cell from the representative experiment shown in Fig 6C, we confirmed that the ZAP that was co-transfected had a negligible effect on the overall myc-TRIM25 protein levels (1x when co-transfected with ZAPL WT vs. 1.08x when co-transfected with ZAPL N658A). The amount of myc-TRIM25 immunoprecipitated by FLAG-ZAP is also apparently equal (1x pulled down by ZAPL WT vs. 1.09x pulled down by ZAPL N658A) (Fig 6C). From all four independent experiments we have performed, we found no statistically significant differences in interaction with TRIM25 between ZAPL WT and ZAPL N658A (Fig 6D), suggesting that the increased antiviral activity of the N658A mutant is not related to changes to its synergy with TRIM25.

We further evaluated whether increased IFN induction is responsible for the enhanced antiviral activity of the ZAPL N658A mutant. After treating ZAPL WT and N658A cell lines with poly(I:C), a double-stranded RNA mimic, to stimulate the IFN response, we performed quantitative polymerase chain reaction (qPCR) analysis of the mRNA levels of IFN-β, IFIT1, and IFIT2, the latter two of which are classical antiviral ISGs. We found that poly(I:C) treatment upregulates IFN-β, IFIT1, and IFIT2 RNA levels, and expression of ZAPL WT and N658A further augments the response (S4 Fig). Importantly, both IFN-β and IFIT1 induction between ZAPL WT and N658A cell lines is similar upon stimulation (S4A and S4B Fig). WT ZAP induces IFIT2 slightly more than N658A ZAP (728x vs. 401x, S4C Fig), but this is in the opposite direction from the superior antiviral activity, as IFIT2 is an antiviral ISG and a higher amount should evoke a more antiviral state. We next asked whether the non-ISG, ZAP-regulated cellular transcript TRAILR4 has distinct RNA levels in ZAPL WT and N658A mutant cell lines. A previous study has shown that siRNA knockdown of ZAP increases TRAILR4 mRNA by about 2.5-fold, and rescue of ZAPL expression by transfection marginally decreases TRAILR4 RNA [13]. We found that inducing ZAP with doxycycline in the absence of poly(I:C) treatment reduced TRAILR4 transcript levels, although the difference is minimal between WT ZAP and N658A (0.9x vs. 0.4x). However, with poly(I:C) treatment to simulate an infected state, TRAILR4 RNA levels are further decreased when ZAPL N658A is expressed (S4D Fig, 1.2x for WT vs. 0.4x for N658A). Taken together, our results rule out a heightened IFN response as responsible for the improved antiviral phenotype of N658A.

### The ZAPL N658A mutant has reduced binding to PAR

**Fig 7:**
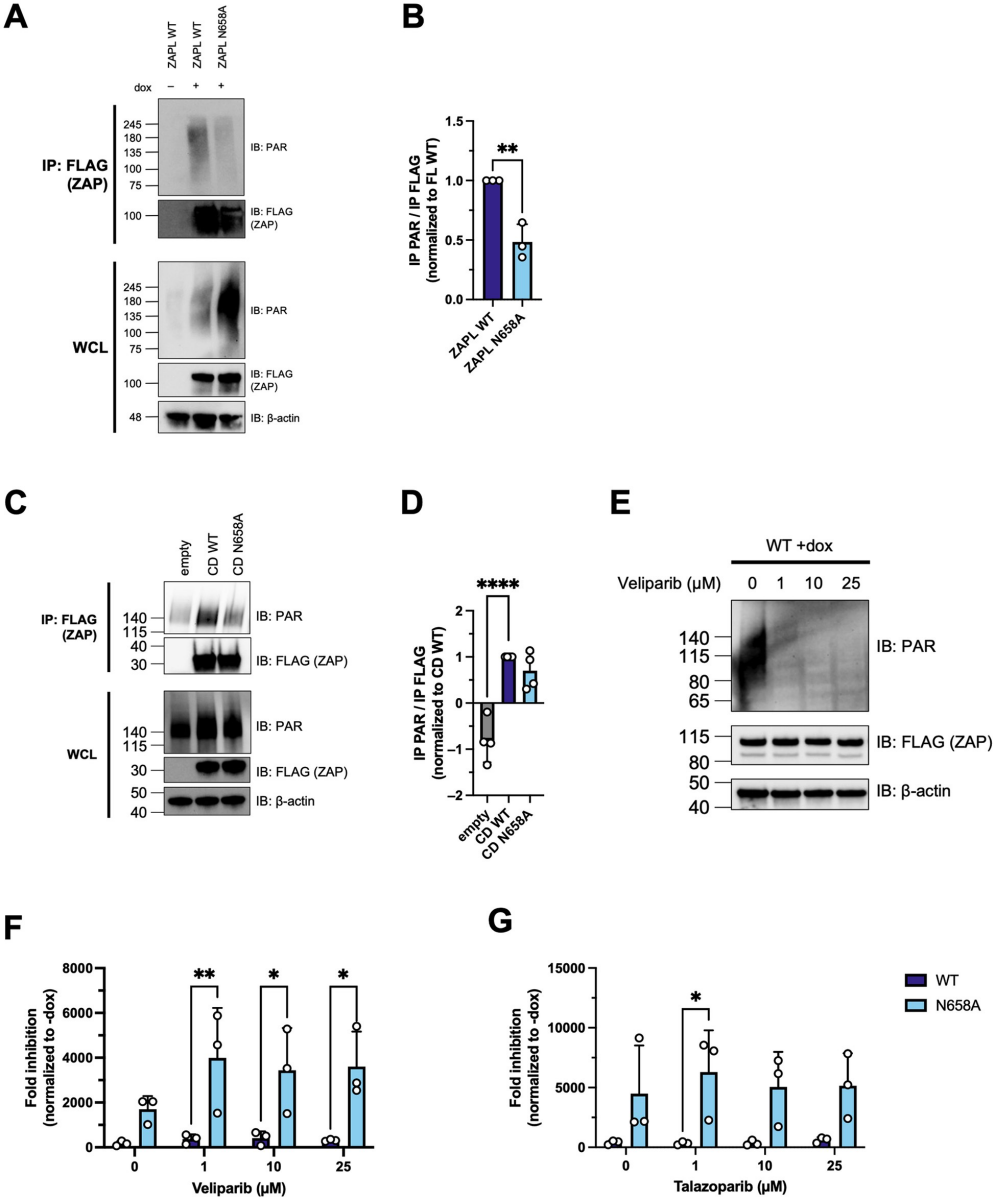
The N658A mutant is correlated with decreased binding to poly(ADP-ribose) (PAR). (A) Western blot of ZAPL WT or N658A in ZAP inducible ZAP KO HEK293T cell lysates immunoprecipitated by FLAG beads after treatment with 1μM PARG inhibitor. Data are representative of three independent experiments. (B) Densitometric analysis of the ratio of PAR pulldown normalized to FLAG-ZAP pulldown as quantified by Image Lab, normalized to WT, and combined from three independent experiments. Error bars indicate standard deviation. Asterisks indicate statistically significant differences as compared to the WT cell line (unpaired t-test: **, p<0.01). (C) Western blot of PAR bound to ZAP in cell lysates of ZAP KO HEK293T transfected with empty pcDNA3.1 vector, pcDNA3.1-3XFLAG-ZAPL-WT, or -N658A central domain. ZAP was immunoprecipitated by FLAG beads after treatment with 1μM PARG inhibitor. Data are representative of three out of four independent experiments. (D) Densitometric analysis of the amount of PAR pulldown normalized to FLAG-ZAP pulldown as quantified by Image Lab, normalized to WT, and combined from four independent experiments. Error bars indicate standard deviation. Asterisks indicate statistically significant differences as compared to the WT cell line (one-way ANOVA and Dunnett’s multiple comparisons test: ****, p<0.0001). (E) ZAPL WT inducible ZAP KO HEK293T cell line treated with 0, 1, 10, and 25μM of the PARP inhibitor Veliparib for 24 hours before harvesting the WCL for western blot. Data is from one experiment. (F, G) ZAPL WT or N658A inducible ZAP KO HEK293T cell line was induced with dox for ZAP expression for 24 hours before infection with SINV Toto1101/Luc at an MOI of 0.01 PFU/cell and treated with 0, 1, 10, and 25μM of the PARP inhibitor Veliparib (F) or Talazoparib (G). The cells were harvested 24 h.p.i for luciferase assay. Fold inhibition is calculated by dividing the averaged -dox RLU by the individual +dox RLU. Data are representative of two independent experiments. Error bars indicate standard deviation. Asterisks indicate statistically significant differences as compared to the corresponding WT cell line (two-way ANOVA and Šídák’s multiple comparisons test: *, p<0.05; **, p<0.0001). 1μg/mL dox is used to induce ZAP expression in ePB ZAP inducible cell lines.

Since RNA binding, TRIM25 interaction, and the IFN response do not appear to mediate the superior antiviral activity of ZAPL N658A, we decided to characterize the effect the mutation has on WWE domain function. The WWE2 in ZAP has recently been found to bind to PAR, an ability that enhances ZAP’s antiviral function against a CpG-enriched HIV-1 [17]. We wondered if mutating site 658, which is within the WWE2, changes ZAP’s ability to bind to PAR. We performed a co-immunoprecipitation assay in which we pulled down ZAP and probed for PAR. PAR levels in the whole cell lysate are markedly lower in cells without ZAP induced (Fig 7A). Compared to ZAPL WT, ZAPL N658A binds to less PAR (Fig 7A). Even though we have seen fluctuating overall PAR levels among independent experiments, the N658A mutant has consistently pulled down less PAR, as demonstrated by our densitometric quantification across three independent experiments (Fig 7B). Altogether, these data suggest that the antiviral activity of this mutant negatively corresponds to ZAPL’s ability to bind PAR, despite the site being outside of the PAR binding groove. The mutation might prevent an active PARP from accessing and PARylating ZAPL in an uninfected cell. Contrary to the Q668R mutation in the PAR binding pocket which diminishes ZAP PAR binding and anti-HIV activity [17], our N658A mutant is less proficient in binding PAR, but surprisingly more adept at restricting SINV.

To further validate the PAR binding phenotype, we included a negative control mutant that had been reported to have attenuated PAR binding (Q668R) [17]. Consistent with previous findings, the Q668R ZAP mutant exhibits a loss in PAR binding activity (S5 Fig). Because the localization of ZAP can change based on the presence of a PARP-like domain, we also tested PAR binding in the context of just the central domain [20,41]. We generated plasmid constructs of FLAG-tagged ZAP central domain with WT and N658A sequences, transfected them into ZAP KO HEK293T cells, and performed a PAR binding assay. Similar to our results in the full-length ZAP context, we found that the central domain N658A mutant is still correlated with less PAR binding than the central domain ZAP WT (Fig 7C) in three out of four independent trials (average ratio of IP PAR/IP FLAG is 0.7x for N658A vs. 1x for WT), although the difference is not statistically significant likely due to the one outlier trial (Fig 7D).

Because PAR binding can be affected by overall PAR levels in the cell, we treated WT and N658A ZAP cell lines with the PARP inhibitors Veliparib and Talazoparib from 1 to 25μM [42–46]. Veliparib is superior in selectively inhibiting PARP1 and PARP2, while Talazoparib inhibits PARP1, PARP2, and tankyrases [47]. We pre-treated the cells with the inhibitors for 1 hour before SINV addition to allow enough time to block PARP activities prior to virus infection, during which we maintained the same concentration of each PARP inhibitor. We harvested the cells for western blot and luciferase assay 24 h.p.i. Consistent with a previous study that used Veliparib [48], PAR is markedly decreased past 1μM of Veliparib treatment (Fig 7E). We found that the antiviral activity of WT ZAP is not enhanced and that the N658A mutant is still more potent than WT ZAP regardless of the concentration of the PARP inhibitor tested (Fig 7F and 7G).

### Asparagine is the predominant amino acid at site 658 in ZAP yet the least antiviral

**Fig 8:**
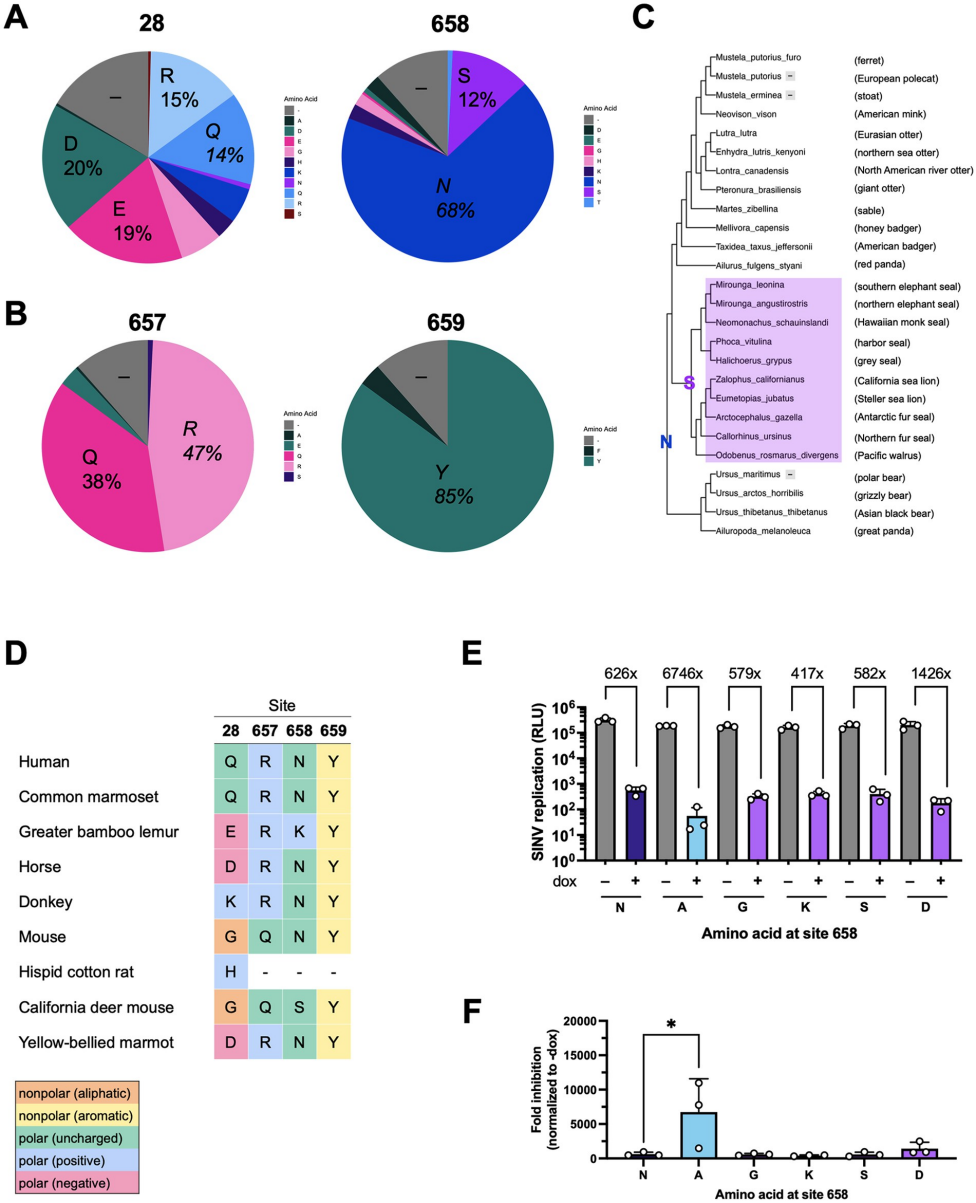
Asparagine is the predominant amino acid at site 658 yet confers weaker antiviral activity. (A, B) The distribution of amino acids at sites 28, 657, 658, and 659. (C) A zoomed in view of the 261-mammals phylogenetic tree showing the nearest relatives of the Pinnipedia clade (seals) and their amino acids at site 658. Gray dashes indicate gaps or deletions. (D) An abridged alignment of amino acids at sites 28, 657, 658, and 659. (E) ZAPL N658 (WT) or N658A/G/K/S/D inducible ZAP KO HEK293T cells were induced for ZAP expression with 1μg/mL dox. Cells were infected with SINV Toto1101/Luc at an MOI of 0.01 PFU/cell and harvested at 24 h.p.i for luciferase assay. Data are representative of two independent experiments. Error bars indicate standard deviation. (F) Fold inhibition of (E) is calculated by dividing the averaged -dox RLU by the individual +dox RLU. Error bars indicate standard deviation. Asterisks indicate statistically significant differences as compared to the corresponding WT cell line (one-way ANOVA and Dunnett’s multiple comparisons test: *, p<0.05).

To further understand the requirements at site 658 for ZAP to become a more potent restrictor, we analyzed the amino acid distribution in our mammalian ZAP sequences. We observed that site 28, one of the positively selected sites, displays an even distribution of amino acids (Fig 8A). In contrast, at site 658, asparagine is the most prevalent amino acid in our 261 mammalian ZAP sequences (68%, Fig 8A). Interestingly, when we looked at what species do not have an asparagine, we found that marine mammals in the Pinnipedia clade all have a serine (Fig 8C). However, there are also other non-pinniped mammals that have a serine, such as the long-tongued fruit bat, Asian palm civet, and the meerkat, suggesting that there is convergent evolution from distinct clades. In terms of the amino acid property, there is less variation at site 658 than at site 28 (Fig 8A and 8D). Even though site 658 has rapidly evolved, polar amino acids seem to be favored by evolution. 80% of the mammals in our alignment have a polar amino acid at site 658: 177 out of the 261 mammals (68%) have asparagine and 32 (12%) have serine (Fig 8A). This is in stark contrast to Q28, where every amino acid property is present: 7% have a nonpolar amino acid (alanine, glycine); 38% have a polar amino acid (glutamine, asparagine, serine); 38% have a negatively charged amino acid (aspartic acid, glutamic acid); and 23% have a positively charged amino acid (histidine, lysine, arginine) (Fig 8A and 8D), demonstrating that site 28 is able to tolerate more flexibility in the chemical property of its amino acid. Adjacent sites that are not under positive selection, 657 and 659, show even less amino acid diversity (Fig 8B). Site 657 is dominated by a polar (glutamine) or positive (arginine) amino acid, and site 659 permits only nonpolar amino acids with an aromatic ring (tyrosine and phenylalanine).

To ascertain if a specific amino acid or a nonpolar property is required at site 658 to achieve better antiviral activity, we generated additional ZAPL N658 mutants by mutating the WT residue in humans, asparagine, to residues found in other mammalian species such as glycine (nonpolar; in African woodland thicket rat), serine (polar uncharged; in California deer mouse), lysine (positive; in greater bamboo lemur), or aspartic acid (negative; in little brown bat). We infected cell lines with inducible expression of each of these ZAPL site 658 mutants with the same luciferase-expressing SINV and found that only the N658A mutant consistently has significantly higher anti-SINV activity than WT ZAP (N658) (Fig 8E and 8F). None of the other naturally occurring residues at site 658 confers significantly more potent activity on ZAP, supporting that ZAP has the potential to be further optimized and improved as a restriction factor. As our findings suggest that the residue at site 658 with the best antiviral activity (N658A) is nonexistent in nature, further studies are required to understand why positive selection has selected for a version of ZAP that does not maximize its anti-alphaviral activity.

## Discussion

In this study, we sought other positively selected sites beyond the three previously identified in the PARP-like domain of ZAP and asked whether they have played a role in response to virus infections. We identified seven positively selected sites in total throughout mammalian evolution of ZAP, with only one residing in the PARP-like domain, supporting the notion that ZAP has been the target in more than one host-virus arms race. Notably, four of these positively selected sites are concentrated in the central region. We found that mutating the positively selected sites did not significantly impair WT ZAP’s original antiviral activity, in line with a deep mutational scanning study of TRIM5α [49]. Interestingly, a mutation at the WWE2 (N658A) was almost 10 times better at inhibiting SINV and other Old World alphaviruses than WT ZAP. Even though an alanine mutation at site 658 is nonexistent in extant mammalian ZAP, our study adds to and is consistent with previous studies on MxA [50,51]. Importantly, just one amino acid change in MxA is sufficient to change its species specificity against an orthomyxovirus [50] and that enhancing mutations do not necessarily have to be naturally occurring [51]. Furthermore, we have been testing other naturally occurring residues at site 658 in the context of human ZAP, although strong effects might require testing these residues in the context of ZAP from their cognate species. Together with our findings, these studies highlight the advantage of positive selection analysis, which facilitates the discovery of improved versions of host antiviral proteins, especially when we are not confined to what is sampled in nature.

Our positive selection analysis incorporates high quality ZAP sequences from all orders of mammals, while most analyses of positive selection in innate immune factors have focused on a subset of species. For example, using 17 primate TRIM5α sequences, Sawyer *et al*. identified five residues under positive selection all within a 13-amino acid patch that is responsible for species specificity against lentiviruses [38]. Enabled by the more comprehensive sequences and robust codon substitution models presently, we hypothesized that including more species would allow us to detect positive selection signatures in regions across the whole protein and provide a more well-rounded picture of antiviral effectors. Consistent with a study that identified distinct positively selected sites in SAMHD1 using different subsets of mammals [52], we found that positively selected sites in ZAP, while concentrated, are not just restricted to the PARP-like domain [15], but span the N-terminus, central region, and C-terminus. This reflects the highly diverse and long evolutionary history of ZAP, which arose during the emergence of tetrapods [24]. Further positive selection analyses in subsets of mammals are required to confirm if each positively selected site or domain is driven by distinct viruses.

We found that mutating the N658 site to alanine in the WWE2 of ZAP creates a ZAP that has stronger anti-alphavirus function, unaltered anti-HIV-1 function, and diminished PAR binding ability. We speculated on why our results are different from a previous study in which it identified a Q668R mutation to have a positive relationship between ZAP’s binding to PAR and anti-HIV-1 activity. First, the Q668 residue is buried in the PAR binding pocket, as opposed to the N658 positively selected residue which is outside of the binding pocket. Second, the previous study found differences in antiviral activity only when a CpG-enriched engineered HIV-1 was used [17], whereas the Q668R mutant has similar antiviral activity as WT ZAP when the HIV-1 tested was not CpG-enriched. Lastly, the effect of PAR binding deficiency might be different between HIV-1 and SINV because they are different viruses with different replication strategies. For instance, ADP-ribosylation may be a post-translational modification exploited by alphaviruses, as a productive alphaviral infection relies on the binding to and removal of ADP-ribose by the highly conserved alphaviral macrodomains encoded by nonstructural protein 3 [53–56]. Thus, ancient HIV-1- and SINV-like viruses have most likely exerted distinct selective pressures on ZAP. Building on the previous study, we recognize that changes in PAR binding may both positively and negatively affect ZAP antiviral activity. In the case of the N658A mutant, we saw that having an alanine is correlated with reduced PAR binding, suggesting that the naturally occurring asparagine residue at this site in human ZAP has maintained relatively higher levels of PAR binding. This can be driven by an evolutionary arms race with PARylated viral proteins. Furthermore, because we can only get a snapshot with extant ZAP sequences, it is not possible to know the directionality of the conflict at this moment in time, i.e. if the asparagine restores recognition of a viral protein, or if a viral protein antagonizes WT ZAP by interacting with the asparagine. On one hand, asparagine could be the “best” version because it is able to balance antiviral activity with other functions of ZAP like PAR binding. On the other hand, the mammals with asparagine might gradually evolve toward a better amino acid in the future. To our surprise, depleting the amount of PAR in the cell with a PARP inhibitor does not change the antiviral activity of WT ZAP, suggesting that PAR binding may be an unintended side effect in the evolutionary arms race, rather than a cause or consequence. Alternatively, decreased PAR binding to the ZAPL N658A mutant may also be a way to reduce PAR-dependent ubiquitination of other proteins that interact with ZAP [57]. In the future, it would be important to carry out more rigorous biochemical assays for PAR binding, such as isothermal titration calorimetry [58] or single-molecule fluorescence resonance energy transfer [59]. This would allow us to formally elucidate the relationship between PAR binding and antiviral activity, as well as the role of macrodomains, PARylation, and/or ubiquitination in virus infection.

Why has evolution selected for an amino acid at site 658 that makes a less antiviral version of mammalian ZAP against alphaviruses? One hypothesis is that catering to a specific virus would limit ZAP’s antiviral activity against another virus. We wondered if our N658A mutant is worse than WT ZAP at inhibiting HIV-1. Consistent with previous studies [15,17,60,61], we found that WT ZAP was only mildly effective against HIV-1 (at best a 2-fold inhibition) and that the N658A mutant had similarly modest anti-HIV activity. Thus, it does not seem that ZAP is in its current form to maintain potency against HIV-1. Since the HIV sensitive to ZAP is an artificially engineered mutant enriched with CpGs in a specific region of the HIV genome, it would be interesting to test our mutant ZAP against this engineered HIV in the context of ZAP sensitivity to CpGs in future studies to determine the impact of the N658A mutation on the breadth of ZAP antiviral activity. Another possibility is that having a stronger antiviral activity incurs a fitness cost on the host cell by interfering with non-immune-related cellular functions of ZAP. In cells not infected by a virus, PAR was bound to ZAP; when cells were treated with arsenite to induce stress granule formation, the amount of PAR on ZAP increased and miRNA-mediated silencing decreased [62]. While the direct mRNA targets bound by ZAP and the miRNA complex remain mostly unknown, ZAP is implicated in the regulation of host transcripts in a non-viral context. For example, the transcript of TRAILR4 transcript, which we found to be modestly downregulated in this study by the ZAPL N658A mutant, is a decoy receptor that is involved in TRAIL-induced apoptosis in cancer [13]. Furthermore, a recent RNA-seq analysis also discovered that ZAPS and ZAPL bind to host mRNAs involved in the unfolded protein response and the epithelial-mesenchymal transition [63]. It would be interesting to explore if any of the cellular pathways that are post-transcriptionally regulated by ZAP are affected by the more antiviral N658A mutation.

ZAP is a broad-spectrum antiviral protein that is effective against members from a wide range of virus families. It is possible that some of our positively selected sites did not have a dramatically better antiviral effect compared to WT ZAP because the selection at these other sites were driven by ancient viruses that were not alphavirus-like. We wonder how our other positive selection mutants would behave against other viruses that infect mammals as their primary reservoir hosts. For instance, alphaviruses and flaviviruses share similar transmission cycles where they circulate between wild mammals and domestic mammalian dead-end hosts. Coronaviruses also commonly exploit mammals as hosts, such as camels for MERS and bats for SARS-CoV-1 [64]. If ancient flavivirus- or coronavirus-like viruses drove the positive selection of ZAP, we expect to see a greater impact on its antiviral activity when ZAP mutants are tested against those viruses. Alternatively, viruses that are not susceptible to the increased antiviral activity of the N658A mutant might encode viral antagonists of ZAP. Notably, we saw that there was no difference in the ability of ZAPL WT and N658A to inhibit VEEV. It is possible that VEEV encodes a viral antagonist that can still recognize ZAP despite the mutation and thus is impervious to any improvement in ZAP’s antiviral activity. Nevertheless, rapid adaptation can happen outside of the context of a pursuer-target relationship with one virus, as long as the mutation confers a fitness advantage. It is just as possible that a host protein engaged in multiple arms races with different viruses would have positively selected sites and residues that affect the outcome in each of these races. This could explain why other naturally occurring residues at site 658 we have already tested were not as effective as N658A because they might only be able to show an effect against other matched virus(es). Our HIV result suggests that ancient retroviruses might not have been the major selective force that led to the positive selection of ZAP throughout mammalian evolution. ZAP was likely engaged in more than one genetic conflict and thus its positively selected sites would have different effects in each of these conflicts. In this case, site 658 appears to be important in the genetic conflict with alphaviruses but not HIV-1. Future studies should identify the viral proteins that are locked in an evolutionary conflict with ZAP and test more viruses from different families.

Lastly, it has been shown that ZAP’s N-terminal domain and TRIM25 from different mammalian species are mostly compatible against CpG-enriched HIV-1 [24]. It is possible for our ZAP mutant to behave differently in the cellular backgrounds of species other than that of humans since the N658A mutation is located outside the N-terminal domain of ZAP, in the central domain. Additional bioinformatic analyses can be done to infer the branches or species that contributed to the signals of positive selection. Future studies that look at the compatibility of the human N658A mutant with the ZAP cofactors expressed by those species will be informative.

Our study is one of the first to look at positive selection of a broad-spectrum antiviral protein in a comprehensive and diverse group of mammals. By understanding what makes a strong restrictor and the host cell constraints, we can design better antiviral therapeutics that have the potential to outrun the virus in the host-virus arms race.

## Materials and methods

### Cell culture

HEK293T (parental and ZAP KO) cells were gifts from Dr. Akinori Takaoka at Hokkaido University [36] and maintained in Dulbecco’s Modified Eagle Medium (DMEM; Thermo Fisher Scientific, Waltham, MA) with 10% fetal bovine serum (FBS; Avantor Seradigm, Radnor, PA). BHK-21 cells (American Type Culture Collection, Manassas, VA) were maintained in Minimal Essential Media (Thermo Fisher Scientific) with 7.5% FBS. 0.1mg/mL poly-L-lysine hydrobromide (Millipore Sigma, Darmstadt, Germany) and water were used to coat cell culture dishes when thawing or seeding each cell line to promote cell adhesion and recovery.

### Plasmids

WT or mutant ZAP was cloned into the plasmid pcDNA3.1-3XFLAG (gift from Dr. Oliver Fregoso, University of California, Los Angeles) as previously described [33]. 3XFLAG-ZAPS and -ZAPL were amplified from the pcDNA3.1-3XFLAG plasmids using primers to add ClaI and NotI restriction sites for ligation into the ePB vector (gift from Dr. Ali Brivanlou, Rockefeller University) [35]. Full-length TRIM25 (gift from Dr. Jae U. Jung at Cleveland Clinic Lerner Research Institute) [65] was cloned into pcDNA3.1-myc as previously described [66]. The ZAP positive selection mutants, PAR binding deficient Q668R mutant, and N658G/K/S/D mutants were generated by the Q5 Site-Directed Mutagenesis Kit (New England Biolabs, Ipswich, MA) or synthesized as a gene block (Twist Bioscience, South San Francisco, CA) with ClaI and NotI restriction sites and ligated into the ePB vector. The ZAP CD WT or N658A mutant in pcDNA was cloned using primers that flanked the CD with restriction sites NotI and XbaI. The identity of all plasmids was confirmed by Sanger (Genewiz/Azenta, South Plainfield, NJ) and whole-plasmid sequencing (Primordium, Monrovia, CA). See S1 File for a list of all primers used in this study.

### Generation of ZAP inducible cell lines

All ZAP inducible cell lines were made via the ePB transposon system in ZAP KO HEK293T cells. Specifically, ZAP KO HEK293T cells were transfected with equal amounts of the transposase plasmid and an ePB transposon vector containing WT or mutant ZAP using X-tremeGENE9 DNA Transfection Reagent (Roche Life Science, Basel, Switzerland) in Opti-MEM (Thermo Fisher Scientific) following manufacturer’s instructions. 1μg/mL puromycin was added 48 hours post-transfection to select for ZAP KO HEK293T cells that have incorporated the ePB transposon. Our ZAPS WT and ZAPL WT cell lines were made by selecting single cell clones that follow two criteria: 1) robustly express ZAP following 24 hours of 1μg/mL doxycycline treatment, and 2) recapitulate differential alphaviral sensitivities (S2 Fig) similar to previously generated bulk cell lines with inducible ZAP expression [7,66]. The mutant ZAP cell lines in this study were bulk cells that survived after puromycin selection. Comparable inducible ZAP expression in each cell line was validated by immunoblotting following treatment with 1μg/mL doxycycline. After the study was completed, we found out that the original ePB-3XFLAG-ZAPL constructs and subsequent positive selection mutant constructs we generated express haplotype 2, while the ZAPS constructs express haplotype 1. Both haplotypes are naturally occurring in human populations and have very similar antiviral activities against the viruses tested in [7]. We decided to investigate the effects of the haplotypes on the positive selection mutants in future studies.

### Viruses and infections

SINV (Toto1101) [67], SINV expressing luciferase (Toto1101/Luc and Toto1101/Luc:ts6) [39], SINV expressing enhanced green fluorescent protein (EGFP) (TE/5’2J/GFP) [68], RRV expressing EGFP (gift from Dr. Mark Heise, University of North Carolina) [69], ONNV expressing EGFP (gift from Dr. Steve Higgs, Kansas State University) [70], CHIKV vaccine strain 181/clone 25 expressing EGFP (gift from Scott Weaver, The University of Texas Medical Branch at Galveston) [70], VEEV vaccine strain TC-83 expressing EGFP (gift from Dr. Ilya Frolov, University of Alabama at Birmingham), and HIV-1 Bru ΔEnv pseudotyped with the glycoprotein from vesicular stomatitis virus have been previously described [8,66,72]. All alphaviral stocks were generated and titered in BHK-21 cells [39]. The amount of virus used for each experiment was determined by the multiplicity of infection (MOI), cell number, and virus titer. HIV-1 stocks were generated as previously described [72] and infection was normalized by units of reverse transcriptase activity [73].

ZAPS/L WT and mutant cell lines were induced for ZAP expression with 1μg/mL of doxycycline 1 day prior to virus infection. To quantify SINV replication, cells were infected with SINV with a luciferase reporter gene (Toto1101/Luc) and harvested 24 h.p.i. To quantify SINV translation, cells were infected with a replication-deficient temperature-sensitive SINV (Toto1101/Luc:ts6) at 37°C for 1 hour to allow virus adsorption, followed by incubation at 40°C and harvested at the specified timepoints. Harvested lysates were measured for luciferase units following manufacturer’s instructions of the Luciferase Assay System (Promega, Madison, WI).

To quantify infection by GFP-alphaviruses, infection was performed as described above and fixed in PBS with 1% FBS and 2% formaldehyde 24 h.p.i. The fixed cells were analyzed on the Attune NxT Flow Cytometer (Thermo Fisher Scientific), courtesy of the UCLA Flow Cytometry Core.

For HIV-1 infection, cells were spinfected at 1200xg for 90 min at 37°C at 7,000 units/mL and 30,000 units/mL of reverse transcriptase activity. Infection was assessed at 24 hours via flow cytometry by an antibody against the HIV-1 core antigen-RD1 (Beckman Coulter) and viability was assessed by Ghost Dye Red 780 (CytekBio).

### Quantification of SINV virion production via plaque assays

To quantify SINV virion production in ZAPL WT or mutant cells, ZAP expression was induced by 1μg/mL doxycycline 1 day prior to infection and infected with SINV Toto1101. The viral supernatant was collected at specific timepoints. To determine viral titers, BHK-21 cells were infected with the viral supernatant at six 10-fold dilutions and incubated at 37°C for 1 hour with gentle rocking every 15 min. Avicel (RC-581 NF, pharm grade, DuPont Nutrition & Health) overlay consisting of 2X MEM and 4.5% Avicel was added to each well and the plate was incubated at 37°C overnight. On the following day, cells were fixed with 7% formaldehyde for 15 min and stained with 1X crystal violet. The plates were washed and the plaques counted after drying.

### Poly(I:C) stimulation, RNA extraction, and reverse transcription quantitative polymerase chain reaction (RT-qPCR)

To stimulate cells with a double-stranded RNA mimic, poly(I:C) diluted in Opti-MEM was incubated with Lipofectamine RNAiMax Transfection Reagent (Thermo Fisher Scientific) before being added to ZAPL WT or mutant cells. 1 day after poly(I:C) stimulation, total RNA was extracted from cells using the Quick-RNA kit (Zymo Research). The amount of RNA template was equalized for reverse transcription using the Protoscript II First Strand cDNA Synthesis Kit and random hexamers (New England Biolabs). RT-qPCR was performed using 10-fold-diluted cDNA and the Luna Universal qPCR Master Mix (New England Biolabs) in the CFX Real-Time PCR system (Bio-Rad), courtesy of the UCLA Virology Core. qPCR conditions were as previously described [66]. Target transcript levels were determined by normalizing the target transcript CT value to the RPS11 transcript CT value. Fold change was calculated using this normalized value relative to that of the corresponding cell line untreated with dox and unstimulated with poly(I:C) (CT method). For RT-qPCR primers, see S1 File.

### Immunoblot analysis

Proteins were visualized using SDS-PAGE with 4–20% Mini-PROTEAN TGX Precast Protein Gels (Bio-Rad) in NuPAGE MOPS SDS Running Buffer (Invitrogen) and transferred to a PVDF membrane (Bio-Rad). The proteins of interest were probed with the corresponding primary and secondary antibodies, followed by visualization on a ChemiDoc imager (Bio-Rad, Hercules, CA) using the ProSignal Pico ECL Reagent detection reagent (Genesee Scientific, El Cajon, CA).

Primary antibody 1:20,000 anti-FLAG (Sigma-Aldrich), 1:20,000 anti-actin-HRP (Sigma-Aldrich), or 1:1000 anti-poly(ADP-ribose) (Abcam); and secondary antibody 1:20,000 goat anti-mouse HRP (Jackson ImmunoResearch, West Grove, PA) or 1:20,000 goat anti-rabbit HRP (Thermo Fisher Scientific) were used to probe the protein of interest.

Band intensity was quantified by Image Lab (Bio-Rad) using Volume Tools and the default local background subtraction method. Detailed description of how the quantification was performed for each experiment can be found in the respective Figure captions.

### *In vitro* biotinylation of SINV RNA and RNA pulldown assays

The genomic SINV DNA template was digested by XhoI and *in vitro* transcribed using SP6 RNA polymerase (New England Biolabs) and 0.5mM biotin-16-UTP (Roche Life Science, Penzberg, Germany) as previously described [33]. RNA biotinylation was confirmed by streptavidin-HRP dot blot as previously described [8].

*In vitro* RNA pulldown was performed as previously described [33]. ZAP expression was induced in ePB ZAP cell lines and the protein lysates were harvested in CHAPS buffer (10mM Tris-HCl pH7.5, 1mM MgCl_2_, 1mM EDTA, 0.5% CHAPS, 10% glycerol, 5mM beta-mercaptoethanol, and protease inhibitor) 24 hours later. 0.4pmol of biotinylated SINV RNA was incubated with normalized amounts of protein lysates and RNA binding buffer containing RNaseOUT (Thermo Fisher), heparin (Sigma-Aldrich), and yeast tRNA (Thermo Fisher) to minimize non-specific binding. The lysate-RNA samples were incubated with Dynabeads M-280 Streptavidin (Invitrogen) on a shaker for 30 min at room temperature. Protein visualization on a ChemiDoc imager was as described above.

### Immunoprecipitation assays

To test interaction with TRIM25, ZAP KO HEK293T cells were transfected with pcDNA3.1-3XFLAG-ZAPL and pcDNA3.1-myc-TRIM25. Cells were lysed in FLAG buffer (100mM Tris HCl pH8.0, 150mM NaCl, 5mM EDTA, 5% glycerol, 0.1% NP-40, 1mM DTT, and protease inhibitor) and incubated on a rotator at 4°C for 30 min. After equilibration, FLAG beads were incubated with lysates on a rotator at 4°C for 45 min. Immunoprecipitated samples were washed three times with FLAG buffer and eluted in Laemmli buffer for immunoblotting.

PAR binding assay was based on [17] with modification. Briefly, ZAP inducible cells, ZAP KO HEK293T cells transfected with ZAP CD plasmids, or cells treated with PARP inhibitors were lysed in lysis buffer containing 50mM Tris-HCl pH7.5, 150mM NaCl, 0.2% Triton X-100, protease inhibitor, and 1μM PARG inhibitor PDD 00017273 (Tocris Bioscience, Bristol, UK). After equilibration, FLAG beads were incubated with lysates on a rotator at 4°C for 1 hour and 30 min. Bound lysates were washed three times with IP buffer (50mM Tris-HCl pH7.5, 150mM NaCl, and 0.2% Triton X-100) and eluted in Laemmli buffer for immunoblotting.

### PARP inhibitor treatment

To block PARP activity, the PARP inhibitors Veliparib (Selleck Chemicals, Houston, TX) and Talazoparib (Selleck Chemicals) were added to cells 1 hour before virus infection and maintained at the same concentration during the 24 hours of infection such that the volume of the diluent (DMSO) did not exceed a 1:1000 dilution in the culture media.

### Sequence alignment, phylogenetic tree, and positive selection analysis

The coding sequence (CDS) of human ZAPXL was used to search for orthologs in 260 other mammalian genome assemblies with a contig size of at least 30kb in the NCBI assembly database as of July 2020 to minimize truncated orthologous coding sequences. To extract the orthologous coding sequences of ZAP, we used best Blat reciprocal hits from the human CDS to every other mammalian genome, and back to the human genome (matching all possible reading frames, minimum identity of 30%, and the “fine” option activated).

The 261 orthologous ZAP were aligned to human ZAPXL with MACSE v2 [74] with maximum accuracy settings (S2 File). The alignments generated by MACSE v2 were then cleaned by HMMcleaner [75] using default parameters to remove errors from genome sequencing and “false exons” that might have been introduced during the Blat search. Visual inspection confirmed that the resulting alignment had a very low number of visibly ambiguous or erroneous segments.

The phylogenetic tree of the 261 mammals was built using IQ-Tree [76] to generate the consensus, maximum likelihood tree with a GTR substitution model with six parameters (GTR-6) which provided the best fit (S2 File). The tree was visualized using the ggtree R package [77].

More complete details on the alignment and phylogenetic tree reconstruction are given in [78] as the same exact pipeline was used for this study.

The positive selection analyses FEL, MEME, and FUBAR were performed using HyPhy from the command line [25–27], with the aforementioned alignment and mammalian tree as inputs. Rodrigue *et al*.’s positive selection test based on a Mutation-Selection balance (Mutselomega) was used as described in [28]. Briefly, Mutation-Selection balance tests attempt to provide higher statistical power to detect positive selection by better accounting for selective constraint in coding sequences, beyond the usual arbitrary use of the dN/dS>1 threshold by other selection tests.

### Statistical analysis

Experiments were performed at least two independent times and statistical analyses were performed on biological replicates from triplicate wells using GraphPad Prism. All graphical presentations have error bars above the plotted bars.

## Supporting information

S1 Fig Positive selection and domains of ZAP. (A) Positive selection analyses on ZAPXL of 261 mammalian species detected by the FEL, MEME, FUBAR, and Rodrigue methods. (B) ZAP isoforms annotated with their domains. The four ZAP splice variants are depicted here: ZAPS (short), ZAPM (medium), ZAPL (long), and ZAPXL (extra-long). All isoforms contain the zinc finger (Z1-Z5, pink) and WWE domains (green), but only ZAPXL and ZAPL have a catalytically inactive PARP-like domain (indigo). ZAPXL and ZAPM also share an extended exon 4 (teal). The amino acid numbering of domains is based on [6,7].
(TIF)

S2 Fig Characterization of WT ZAP inducible single clone cell lines. (A) Western blot of ZAPS and ZAPL WT inducible ZAP KO HEK293T cell lysates. Each single clone cell line was treated with dilutions of dox 24 hours after seeding. Cell lysates were harvested 24 hours after dox treatment. (B) ZAPS and ZAPL WT inducible ZAP KO HEK293T cells were induced for ZAP expression 24 hours before infection by GFP-expressing alphaviruses and harvested at the time listed for flow cytometry (SINV, MOI = 10, harvest 8 h.p.i.; RRV, MOI = 10, harvest 24 h.p.i.; ONNV, MOI = 0.1, harvest 18 h.p.i.). Data are representative of two independent experiments. Error bars indicate standard deviation.
(TIF)

S3 Fig Densitometric analysis of ZAP positive selection mutants. Densitometric analysis on the western blot of ZAPS (A) and ZAPL (B) positive selection mutants as shown in Fig 2A and 2D. The band intensity of FLAG was divided by the band intensity of β-actin for all +dox samples, and the ratios were normalized to that of the corresponding WT ZAP.
(TIF)

S4 Fig N658A mutant induces interferon (IFN) and interferon-stimulated gene (ISG) levels similar to WT. ZAPL WT or N658A inducible ZAP KO HEK293T cells were untreated, treated with poly(I:C), or treated with both poly(I:C) and dox. RNA was harvested for RT-qPCR. mRNA levels of IFN-β (A), the ISGs IFIT1 (B) and IFIT2 (C), and TRAILR4 (D) in each condition were normalized to that of the respective cell line without poly(I:C) and without dox. Data are representative of two independent experiments. Asterisks indicate statistically significant differences as compared to every other condition and to each cell line (two-way ANOVA and Tukey’s multiple comparisons test: *, p<0.05; **; p<0.01; ***, p<0.001; ****, p<0.0001).
(TIF)

S5 Fig The ZAPL PAR binding deficient Q668R negative control pulls down less PAR. Western blot of ZAP KO HEK293T cells, ZAPL Q668R, WT, and N658A inducible ZAP KO HEK293T cell lysates are immunoprecipitated by FLAG beads after treatment with 1μM PARG inhibitor. Data are representative of two independent experiments.
(TIF)

S1 File Primers used in this study. (XLSX)

S2 File Alignment and phylogenetic tree from the 261 mammalian ZAP sequences. (ZIP)

S3 File All raw data used in statistical analyses and graphs. (ZIP)
