# Supplementary figures and images for: Positive selection analyses identify a single WWE domain residue that shapes ZAP into a more potent restriction factor against alphaviruses
Source: PLoS Pathog. 2024 Aug 29;20(8):e1011836. doi: 10.1371/journal.ppat.1011836 (PMC11361444; doi:10.1371/journal.ppat.1011836)

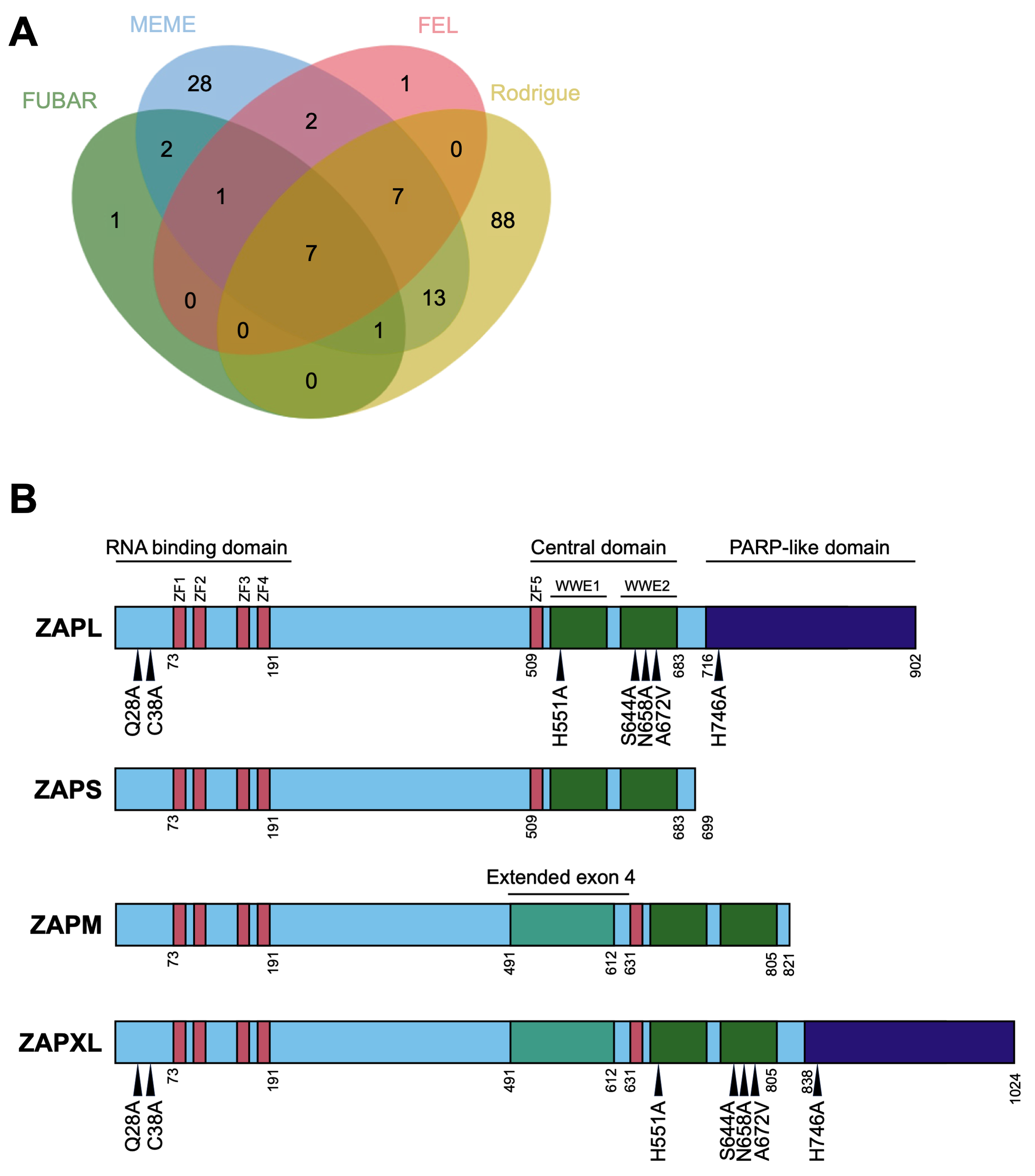

Supplement: S1 Fig — (A) Positive selection analyses on ZAPXL of 261 mammalian species detected by the FEL, MEME, FUBAR, and Rodrigue methods. (B) ZAP isoforms annotated with their domains. The four ZAP splice variants are depicted here: ZAPS (short), ZAPM (medium), ZAPL (long), and ZAPXL (extra-long). All isoforms contain the zinc finger (Z1-Z5, pink) and WWE domains (green), but only ZAPXL and ZAPL have a catalytically inactive PARP-like domain (indigo). ZAPXL and ZAPM also share an extended exon 4 (teal). The amino acid numbering of domains is based on [6,7]. (TIF) [file ppat.1011836.s001.tif]

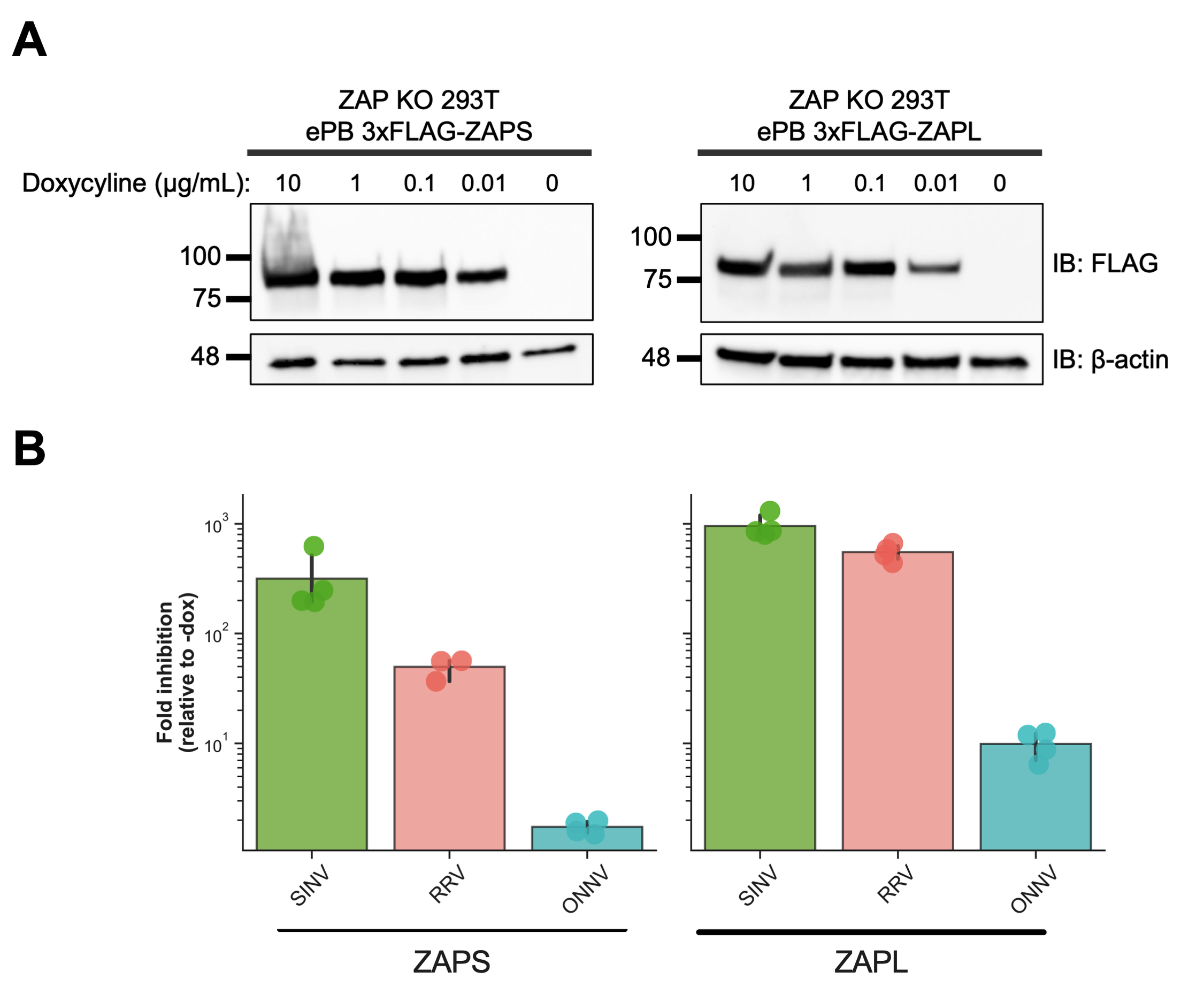

Supplement: S2 Fig — (A) Western blot of ZAPS and ZAPL WT inducible ZAP KO HEK293T cell lysates. Each single clone cell line was treated with dilutions of dox 24 hours after seeding. Cell lysates were harvested 24 hours after dox treatment. (B) ZAPS and ZAPL WT inducible ZAP KO HEK293T cells were induced for ZAP expression 24 hours before infection by GFP-expressing alphaviruses and harvested at the time listed for flow cytometry (SINV, MOI = 10, harvest 8 h.p.i.; RRV, MOI = 10, harvest 24 h.p.i.; ONNV, MOI = 0.1, harvest 18 h.p.i.). Data are representative of two independent experiments. Error bars indicate standard deviation. (TIF) [file ppat.1011836.s002.tif]

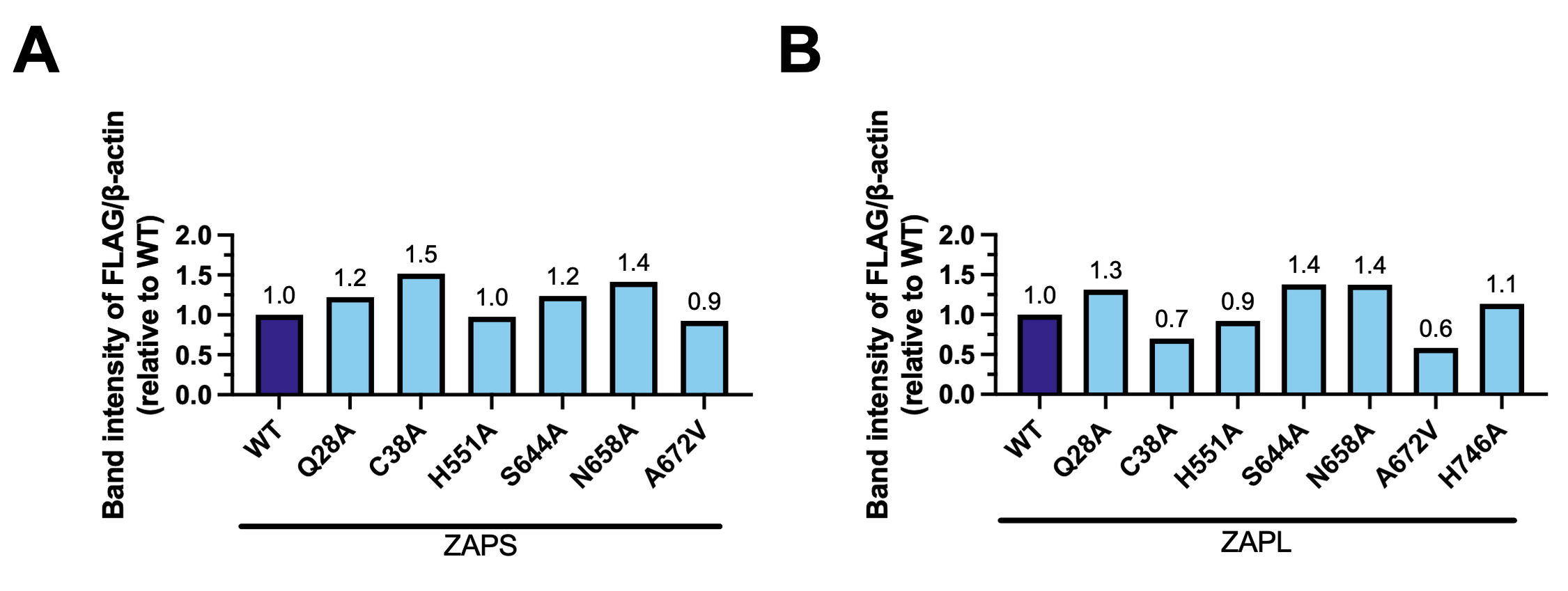

Supplement: S3 Fig — Densitometric analysis on the western blot of ZAPS (A) and ZAPL (B) positive selection mutants as shown in Fig 2A and 2D. The band intensity of FLAG was divided by the band intensity of β-actin for all +dox samples, and the ratios were normalized to that of the corresponding WT ZAP. (TIF) [file ppat.1011836.s003.tif]

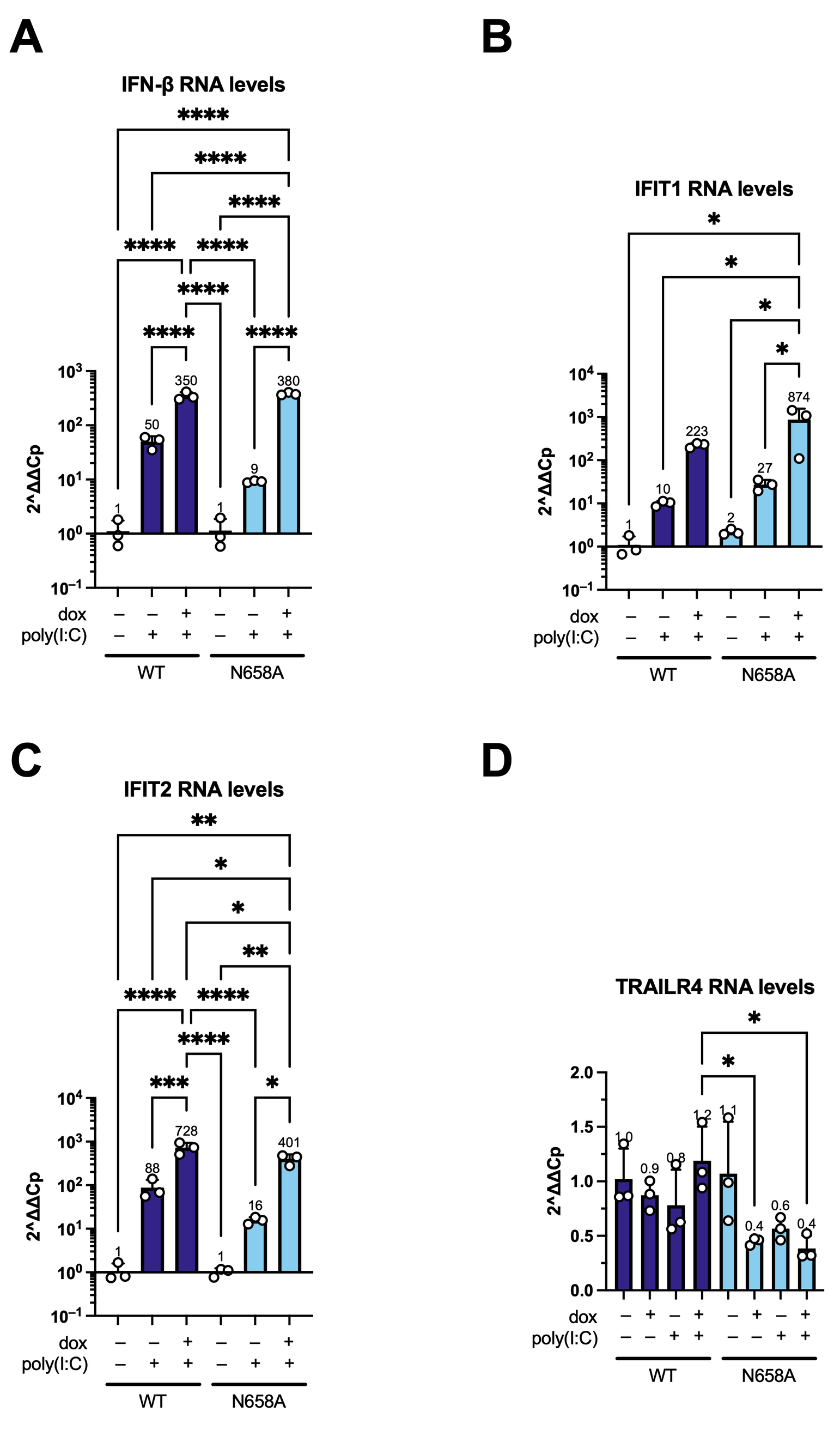

Supplement: S4 Fig — ZAPL WT or N658A inducible ZAP KO HEK293T cells were untreated, treated with poly(I:C), or treated with both poly(I:C) and dox. RNA was harvested for RT-qPCR. mRNA levels of IFN-β (A), the ISGs IFIT1 (B) and IFIT2 (C), and TRAILR4 (D) in each condition were normalized to that of the respective cell line without poly(I:C) and without dox. Data are representative of two independent experiments. Asterisks indicate statistically significant differences as compared to every other condition and to each cell line (two-way ANOVA and Tukey’s multiple comparisons test: *, p<0.05; **; p<0.01; ***, p<0.001; ****, p<0.0001). (TIF) [file ppat.1011836.s004.tif]

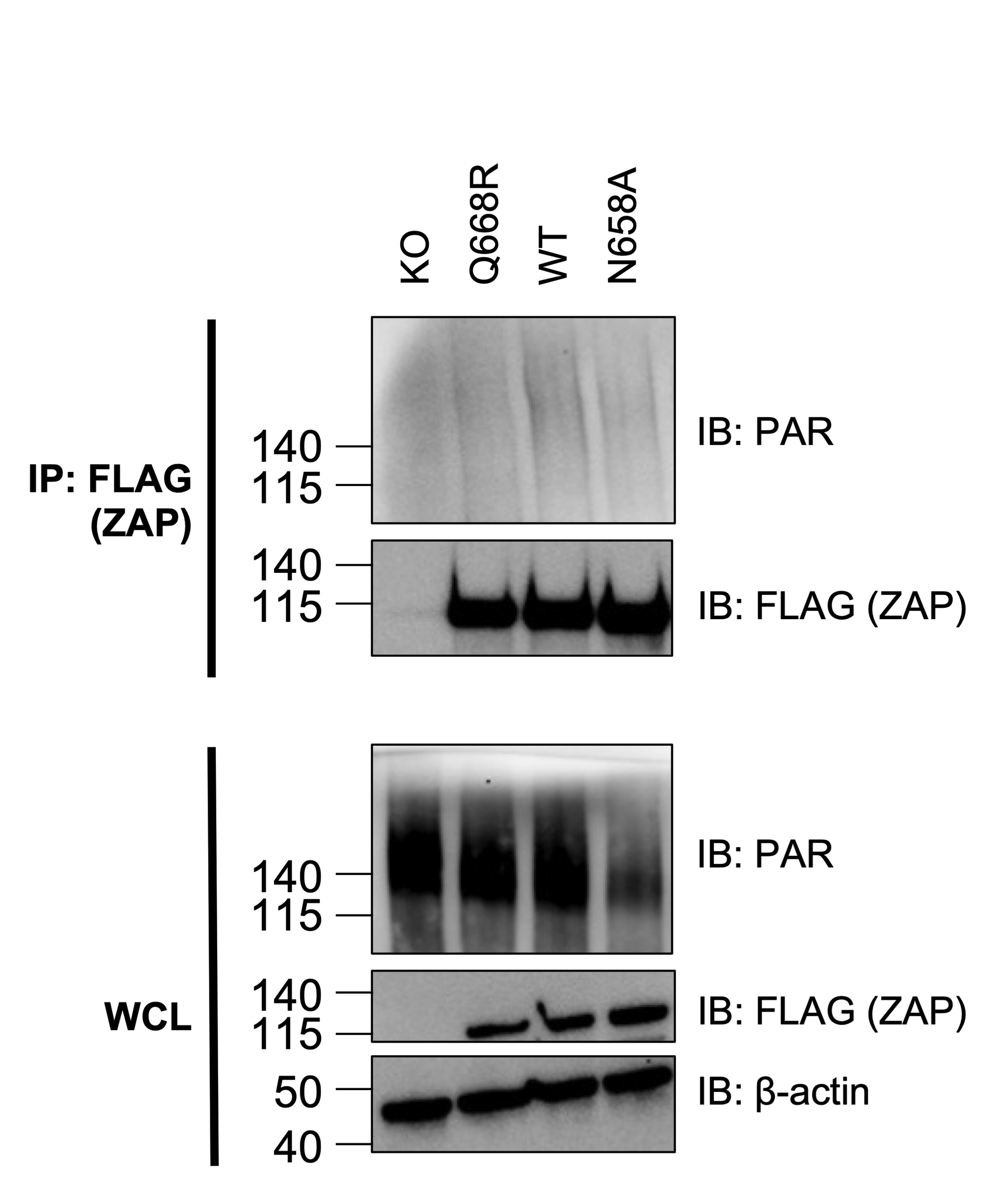

Supplement: S5 Fig — Western blot of ZAP KO HEK293T cells, ZAPL Q668R, WT, and N658A inducible ZAP KO HEK293T cell lysates are immunoprecipitated by FLAG beads after treatment with 1μM PARG inhibitor. Data are representative of two independent experiments. (TIF) [file ppat.1011836.s005.tif]
